# Causal association of juvenile idiopathic arthritis or JIA-associated uveitis and gut microbiota: a bidirectional two-sample Mendelian randomisation study

**DOI:** 10.3389/fimmu.2024.1356414

**Published:** 2024-07-24

**Authors:** Jun-bin Hong, Yue-xuan Chen, Zhi-ying Su, Xin-ying Chen, Yan-ni Lai, Jing-hua Yang

**Affiliations:** ^1^ Department of Pediatrics, The Second Affiliated Hospital, Guangzhou University of Chinese Medicine, Guangzhou, China; ^2^ Shenzhen Hospital of Guangzhou University of Chinese Medicine (Futian), Shenzhen, China; ^3^ School of Medicine and Health, Shunde Polytechnic, Foshan, China; ^4^ Xiaorong Luo’s National Renowned Expert Inheritance Studio, Guangdong Provincial Hospital of Chinese Medicine, Guangzhou, China

**Keywords:** juvenile idiopathic arthritis, uveitis, gut microbiota, causality, bidirectional, Mendelian randomisation analysis

## Abstract

**Background:**

The gut microbiota significantly influences the onset and progression of juvenile idiopathic arthritis (JIA) and associated uveitis (JIAU); however, the causality remains unclear. This study aims to establish a causal link between gut microbiota and JIA or JIAU.

**Methods:**

Using publicly available genome-wide association studies (GAWS) summary data, we conducted a two-sample Mendelian randomisation (MR) analysis employing various methods, namely inverse variance weighted (IVW), simple mode, weighted mode, weighted median and MR-Egger regression methods, to assess the causal association between JIA or JIAU and gut microbiota. Sensitivity analyses, including Cochrane’s Q test, MR-Egger intercept test, leave-one-out analysis and MR-PRESSO, were performed to evaluate the robustness of the MR results. Subsequently, reverse MR analysis was conducted to determine causality between gene-predicted gut microbiota abundance and JIA or JIAU.

**Results:**

The MR analysis revealed a causal association between gut microbiota abundance variations and JIA or JIAU risk. Specifically, the increased abundance of genus *Ruminococcaceae UCG013* (OR: 0.055, 95%CI: 0.006–0.103, *p* = 0.026) and genus *Ruminococcaceae UCG00*3 (*β*: 0.06, 95%CI: 0.003–0.117, *p* = 0.041) correlated with an increased risk of JIA, while genus *Lachnospiraceae UCG001* (OR: 0.833, 95%CI: 0.699~0.993, *p* = 0.042) was associated with a reduced risk of JIA, among others. Sensitivity analysis confirmed MR analysis robustness.

**Conclusions:**

This study provides substantial evidence supporting a causal association between genetically predicted gut microbiota and JIA or JIAU. It highlights the significant role of intestinal flora in JIA or JIAU development, suggesting their potential as novel biomarkers for diagnosis and prevention. These findings offer valuable insights to mitigate the impact of JIA or JIAU.

## Introduction

Juvenile idiopathic arthritis (JIA) is a heterogeneous condition characterised by arthritis of unknown origin manifesting before the age of 16 (1), often resulting in functional limitations and, in severe cases, disability (2). Globally, approximately three million children are affected by JIA (3, 4), making it the most prevalent chronic inflammatory rheumatic disease in childhood. Severe cases may exhibit persistent systemic symptoms, joint inflammation, severe drug side effects and macrophage activation syndrome (MAS) occurrence, which can pose life-threatening consequences, thereby placing a considerable burden on children’s health and socioeconomic systems (5). Juvenile idiopathic arthritis-associated uveitis (JIAU) is commonly acknowledged as a prevalent and severe extra-articular manifestation of JIA (6), characterised by chronic bilateral recurrent anterior uveitis, a leading cause of disability and visual impairment. The incidence of JIAU varies from 5.0% to 19.1% among JIA populations across different geographical regions (7). Although multiple factors, such as genetics, environment and immunity, are hypothesised to contribute to the pathogenesis and underlying mechanisms of JIA and JIAU (6, 8), their precise aetiology and pathogenesis remain unclear, necessitating further investigation to enhance diagnosis, treatment and reduce associated disease burden. The human intestinal microbiota, comprising approximately 100 trillion bacteria of 1000–1150 species, forms a symbiotic relationship with the host, playing critical roles in human metabolism, nutrient absorption, immune responses and other aspects (9). Additionally, the intestinal microbiota is intricately linked to the onset and progression of various human diseases. In the context of predictive, preventive and personalised medicine, systemic inflammation serves as an essential communication bridge between the human host and the gut microbiota (10). Recent evidence suggests a potential role of gut microbiota in immune-mediated diseases such as rheumatoid arthritis (RA), wherein dysbiosis of the gut microbiota disrupts intestinal barrier function, leading to increased permeability and immune imbalance. Consequently, this dysregulation allows immune cells to migrate to extraintestinal sites, including joints, thereby triggering localised inflammation (11, 12).

Recent case-control studies spanning three continents provide compelling evidence of the influence of gut microbiota dysbiosis on the onset and progression of JIA (13–16). A prospective study focussing on the gut microbiota in patients with JIA in Italy and the Netherlands reports the presence of gut microbial dysbiosis in this population. Moreover, significant differences in microbial diversity and composition exist between patients with JIA and healthy individuals (17). Another study conducted in China yielded similar findings, indicating that patients with JIA display a significantly lower abundance of *Anaerostipes, Dorea, Lachnospira* and *Roseburia* compared to the control group. The study also identified 12 genera that could potentially serve as biomarkers and predictive factors for JIA (18). Nevertheless, the causal relationship between JIA and the gut microbiota remains uncertain, necessitating further investigation. Furthermore, observational studies are prone to the impact of confounding variables and reverse causality, potentially biasing results (19).

To address these limitations, we employ Mendelian randomisation (MR) to investigate the genetic-level association between JIA or JIAU and gut microbiota. MR utilises single nucleotide polymorphisms (SNPs) as instrumental variables (IVs) to estimate potential causal links between exposure variables and health outcomes (20). Leveraging extensive genome-wide association studies (GWAS) data, we employ a bidirectional two-sample MR approach to explore the causal relationship between the gut microbiota and these diseases, offering novel perspectives into potential therapeutic strategies targeting the microbiota (21–23).

## Methods

### Description of the research design

**Figure 1:**
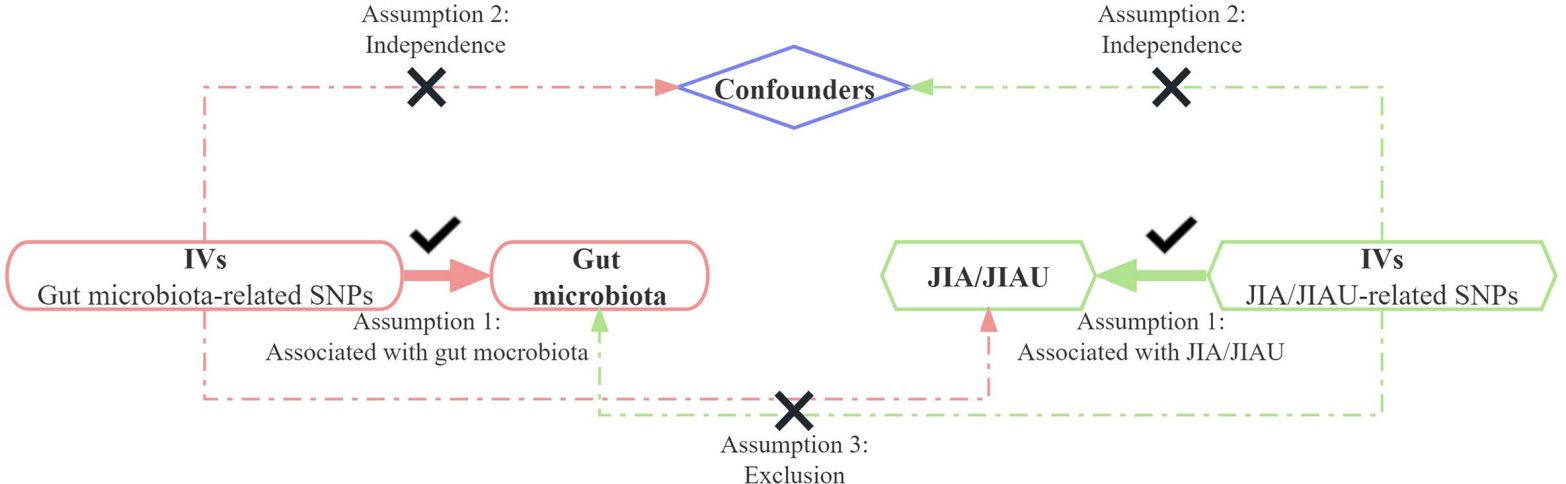
Bidirectional two-sample Mendelian randomisation between JIA/JIAU and gut microbiota abundance outcomes. Directed acyclic graph (DAG) of the causal association between JIA/JIAU and gut microbiota abundance. IVs, instrumental variables; SNPs, single nucleotide polymorphisms; JIA, juvenile idiopathic arthritis; JIAU, JIA-associated uveitis.

**Figure 2:**
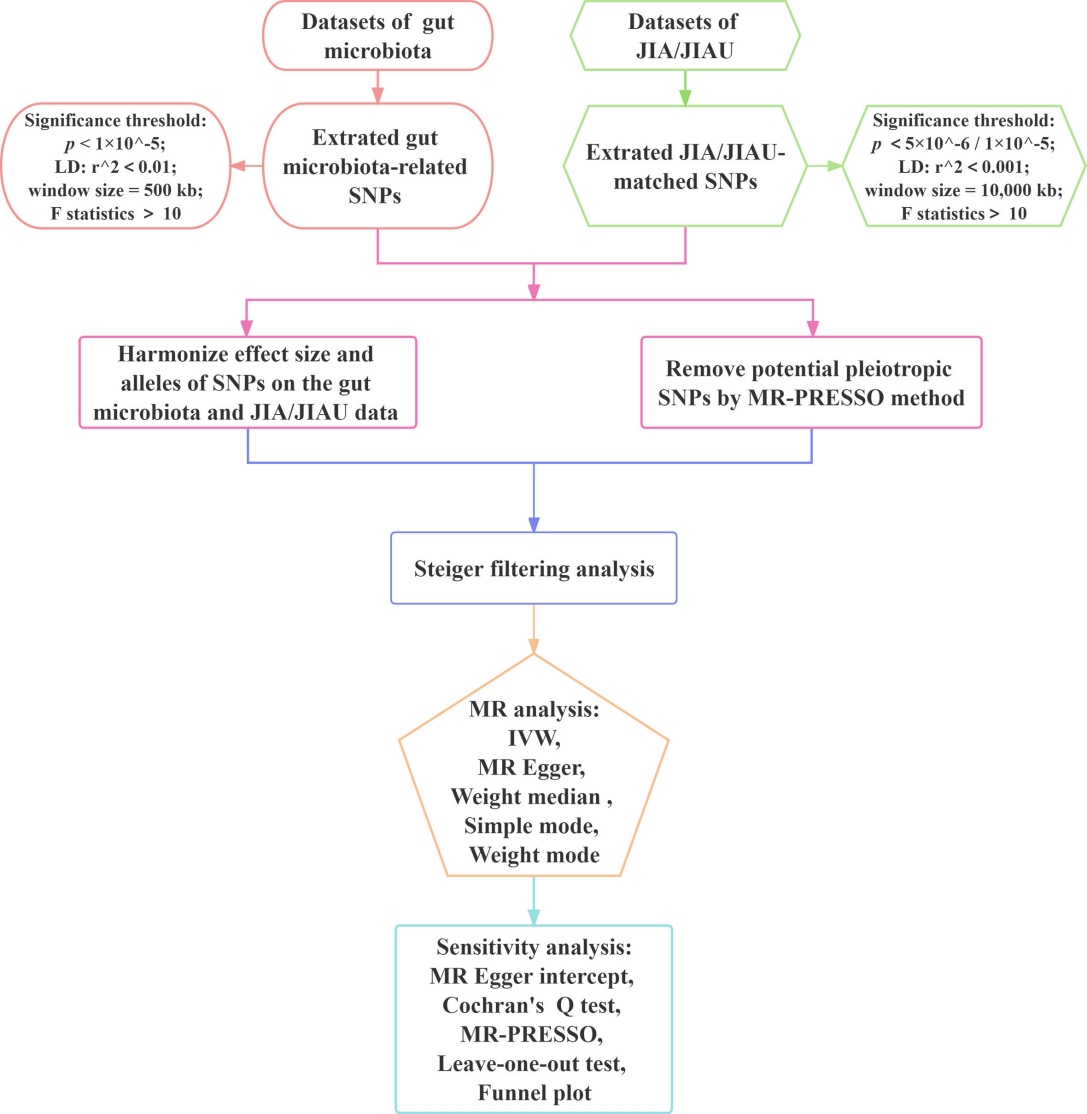
Workflow of Mendelian randomisation study revealing causality between gut microbiota abundance and JIA/JIAU risk. JIA, juvenile idiopathic arthritis; JIAU, JIA-associated uveitis; SNPs, single nucleotide polymorphisms; LD, linkage disequilibrium; IVW, inverse variance weighted; MR, Mendelian randomisation; MR-PRESSO, MR pleiotropy residual sum and outlier.

This study employs a bidirectional two-sample MR approach to evaluate the causal associations between the genetic prediction of JIA or JIAU and the genetic prediction of gut microbiota. Three key assumptions guide the selection of SNPs as IVs: 1) relevance, requiring a substantial correlation between the SNP and the exposure variable; 2) exclusion restriction, indicating that the SNP influences the outcome solely through the exposure variable and not by any alternative causal pathway; and 3) independence, implying that the SNP is independent of the outcome variable and potential confounding factors. In forward MR analysis, gut microbiota serves as the exposure variable, while JIA or JIAU is considered the outcome variable. Conversely, in reverse MR analysis, JIA or JIAU is regarded as the exposure variable, while gut microbiota is treated as the outcome variable. This approach aims to investigate potential causal links between JIA or JIAU and gut microbiota in both directions. **Figure 1** illustrates the schematic representation of the MR causality research design, elucidating the underlying principles of MR studies. **Figure 2** presents a flowchart outlining the step-by-step process involved in conducting such a study. This study adheres to the reporting guidelines outlined in STROBE-MR, supplemented with materials that include a checklist according to STROBE-MR and a checklist based on the Critical Appraisal Checklist for examining MR research (24, 25). The **Supplementary Materials** provide a detailed explanation of the checklist items.

### Data source

#### JIA and JIAU

GWAS data for JIA are based on a recent meta-analysis comprising 6,056 patients with JIA and 25,086 European ancestry controls (26). The detailed research process is described in previous studies by Lopez-Isac E and McIntosh LA (26, 27). The diagnostic criteria for JIA follow international standards published by the International League of Associations for Rheumatology (ILAR), with the specificity and common susceptibility loci for the various JIA ILAR subtypes systematically examined using the JIA GWAS data. The GWAS data for JIAU were sourced from Haasnoot et al.’s study (28), encompassing 192 patients with JIAU of European ancestry and 330 JIA control patients without uveitis.

### Gut microbiota

GWAS data related to gut microbiota were obtained from the Mi BioGen consortium, representing the most extensive dataset available (https://mibiogen.gcc.rug.nl/). The GWAS dataset includes 16S rRNA gene information from the faecal microbiomes of 18,340 individuals across 24 cohorts, alongside whole-genome genotyping data. Specifically, the study investigated the species composition of gut microbiota utilising the 16S rRNA gene regions V1-V2, V3-V4 and V4, representing three distinct variable regions. The dataset comprises 211 intestinal flora, categorised by kingdom, phylum, order, family, genus and species. These species exhibit relative abundance or genetic variations and can be further categorised into 9 phyla, 16 classes, 20 orders, 35 families and 131 genera. After excluding 12 unknown genera, we incorporated a total of 119 genera as classification units for the bidirectional MR study. **Supplementary Table 1** presents the features of the data sources utilised in this investigation.

### The selection of IVs

To ensure the validity of the causal relationship between gut microbiota composition and JIA or JIAU risk, rigorous quality control measures were applied in selecting appropriate genetic IVs.

Given the limited number of genetic variants associated with gut microbiota, we established a significance threshold of *p<* 1.0×10^-5^ based on the majority of MR studies on gut microbiota to ensure adequate candidate instrument numbers for forward MR analysis (29–31). Additionally, to ensure independence between IVs and avoid bias from linkage disequilibrium (LD) between SNPs, we applied LD clustering with a cutoff of *r^2^* < 0.01 and a clustering distance of 500 kb. Finally, to minimise instrument bias, IVs with an *F* statistic less than 10 were excluded, where $F=\left(\text{N}-2\right)\times \frac{R^{2}}{1-R^{2}}$ , with N representing the sample size and *R^2^* the squared correlation coefficient (32).

For reverse MR, the significance threshold was set at *p*< 5.0×10^-6^ to screen SNPs as IVs for JIA and *p<* 1.0×10^-5^ for JIAU, with LD set at *r^2^* < 0.001 and a clumping distance of 10,000 kb.

To determine the direction of influence of specific IVs on the outcome, Steiger filtering analysis was employed (33). IVs with a ‘TRUE’ result indicating the expected association direction were included in subsequent analysis, with those classified as ‘False’ were excluded.

### Statistics analysis

MR analysis was conducted to explore the causality between JIA/JIAU and gut microbiota using R software version 4.2.2 and three specific R packages: ‘TwoSampleMR’ (v.0.5.6) (33), ‘MRPRESSO’ (v.1.0) (34) and ‘MendelianRandomization’ (35). A significance threshold of *p*< 0.05 was considered for causation. To account for multiple comparisons at every taxonomic level (phylum, class, order, family and genus), a significance threshold of 0.05/n was set, where n represents the number of distinct bacterial species at that particular taxonomic level.

Five statistical methodologies were employed: inverse variance weighted (IVW), simple mode, weight mode, weighted median and MR-Egger regression. IVW, considered the primary method, was used for outcome determination, supplemented by the other four methods. Different methods possess different underlying assumptions about horizontal pleiotropy. IVW employs the inverse of the squared standard error (SE) of the outcome as weights and does not include an intercept component in regression. Additionally, random effects were utilised for IVW modelling, with overall estimates derived from weighted linear regression of the Wald estimate for each SNP (36). The other four methods complement IVW by providing more robust estimates in broader scenarios, albeit with lower effect values (wider CI) (34). Moreover, the MR-Egger method offers a consistent causal effect test when SNPs directly linked with the outcome or exhibiting horizontal pleiotropy are excluded (37). Weighted median methodology can produce reliable estimates even when up to 50% of findings are from erroneous SNPs (38). Meanwhile, the simple mode provides an unweighted mode of estimating causal effects’ empirical density function (39). **Supplementary Table 2** lists the advantages, disadvantages, efficacy and applications of these five methods.

### Sensitivity analysis

Following MR analysis, sensitivity analysis was conducted to assess the robustness of the results. Firstly, the intercept of the MR-Egger regression was examined to detect the presence of horizontal pleiotropy, with *p* > 0.05 suggesting a weaker potential for pleiotropy in the causal analysis (34). Additionally, MR-PRESSO was employed to identify and address possible outliers by screening for heterogeneity and outliers, followed by a re-analysis of the MR. To assess heterogeneity in the IVW method, Cochran’s Q test (40) and funnel plots were used. Furthermore, to evaluate the impact of individual SNPs on the primary causal relationship, a leave-one-out analysis was conducted by sequentially excluding individual SNPs.

### Approval, registration and consent

All GWAS data utilised in our analysis were obtained from publicly available datasets with ethical permission granted by the relevant ethical review boards. These datasets do not contain personal information, ensuring compliance with ethical standards.

## Results

### Forward MR

In the initial steps, a total of 3036 SNPs relevant to gut microbiota at the phylum, class, order, family and genus levels were identified, each with an *F* statistic > 10, indicating minimal instrumental bias (**Supplementary Table 2**).

Additionally, Steiger filtering analysis revealed no SNPs with a reverse causal direction for gut microbiota on JIA (**Supplementary Table 3**), while 849 SNPs were eliminated for JIAU (**Supplementary Table 5**). For our analysis, a total of 131 genera, 35 families, 16 classes, 20 orders and 9 phyla were identified as IVs, with 5 to 26 IVs obtained from each classification.

To address multiple comparisons, significance thresholds for gut microbiota on JIA and JIAU were determined using the Bonferroni correction: For JIA, thresholds were set at phyla (*p* = 5.56 × 10^-3^), class (*p* = 3.13 × 10^-3^), order (*p* = 2.50 × 10^-3^), family (*p* = 1.35 × 10^-3^) and genus (*p* = 3.82× 10^-4^). Similarly, for JIAU, thresholds were set at phyla (p = 5.56 × 10^-3^), class (*p* = 3.13 × 10^-3^), order (*p* = 2.94 × 10^-3^), family (*p* = 1.43 × 10^-3^) and genus *(p* = 3.82× 10^-4^).

**Figure 3:**
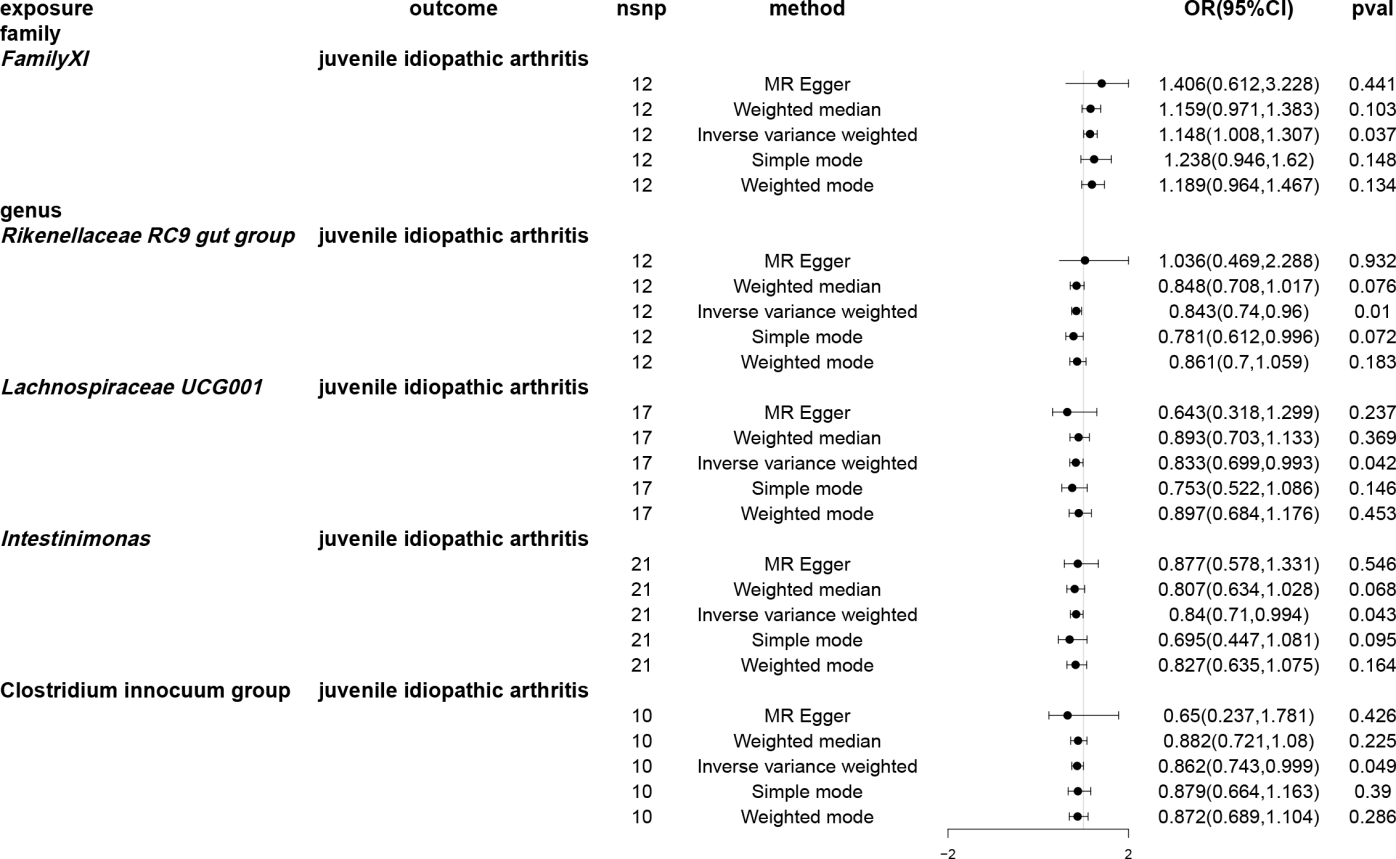
Forest plots of Mendelian randomisation for two samples indicated the causal effects of five different Mendelian randomisation methods with gut microbiota abundance as exposure and juvenile idiopathic arthritis (JIA) as the outcome. The effect estimates are presented as the effect size (OR) and 95% confidence interval (CI). snp, single nucleotide polymorphism.

**Figure 4:**
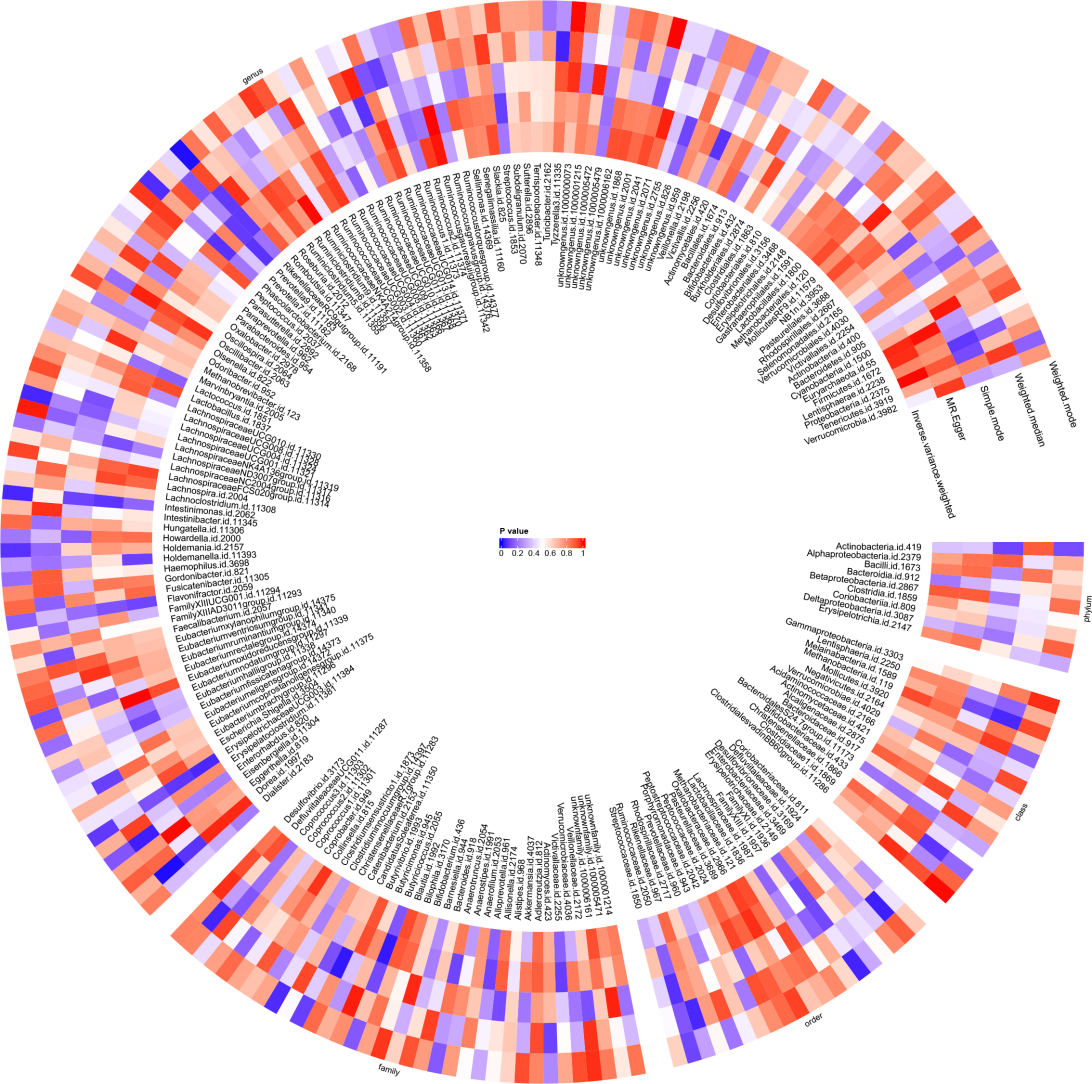
Circular heat map was generated to visualise the results of Mendelian randomisation analysis conducted on two sample groups, illustrating the causal effects estimated by five different Mendelian randomisation methods, with gut microbiota abundance as the exposure variable and juvenile idiopathic arthritis (JIA) as the outcome variable.

Utilising the IVW method, an association between JIA risk and gut microbiota at one phylum and four genera was identified (**Figure 3**). MR analysis revealed that family *FamilyXI* (OR: 1.148, 95%CI: 1.008~1.307, *p* = 0.037) increased the risk of JIA, while genera *Rikenellaceae RC9* gut group (OR: 0.843, 95%CI: 0.74~0.96, *p* = 0.01), *Lachnospiraceae UCG001* (OR: 0.833, 95%CI: 0.699~0.993, *p* = 0.042), *Intestinimonas* (OR: 0.840, 95%CI: 0.709~0.994, *p* = 0.0426) and *Clostridium innocuum group* (OR: 0.862, 95%CI: 0.73~1.0, *p* = 0.0495) decreased the risk of JIA. Notably, consistent effect directions were observed for family *FamilyXI*, genus *Lachnospiraceae UCG001*, genus *Intestinimonas*, and genus *Clostridium innocuum group* across multiple MR methods. However, for the genus *Rikenellaceae RC9* gut group, the results from the MR-Egger method differed from the IVW method but remained consistent with the weighted median, simple mode, and weighted mode methods. A circular heat map visualised the outcomes of MR analysis conducted on two sample groups, illustrating the causal effects estimated by the five different MR methods, with gut microbiota abundance as the exposure variable and JIA as the outcome variable (**Figure 4**). **Supplementary Figures 1**, **2** display the scatter plot and forest plot, respectively.

**Figure 5:**
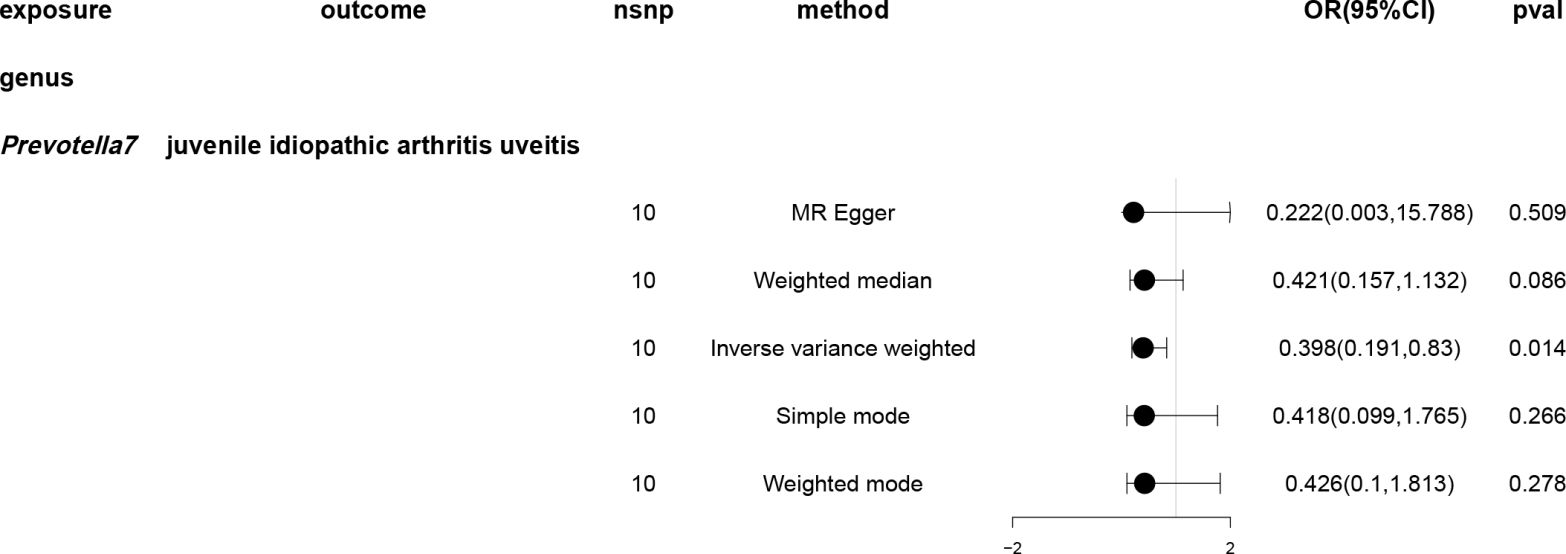
Forest plots of Mendelian randomisation for two samples demonstrated the causal effects of five different Mendelian randomisation methods with gut microbiota abundance as exposure and juvenile idiopathic arthritis associated uveitis (JIAU) as the outcome. The effect estimates are presented as the effect size (OR) and 95% confidence interval (CI). snp, single nucleotide polymorphism.

**Figure 6:**
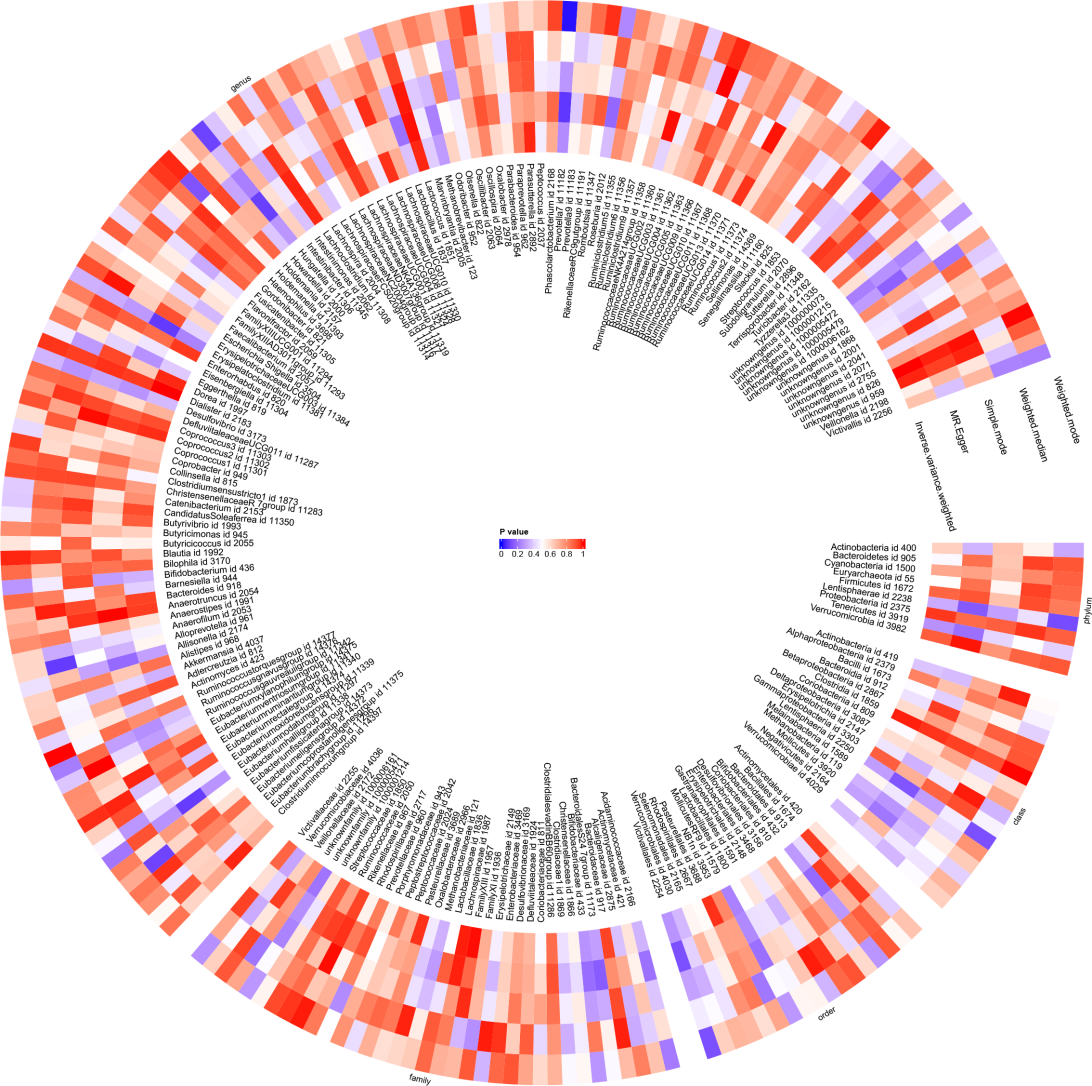
With gut microbiota abundance as the exposure variable and juvenile idiopathic arthritis associated uveitis (JIAU) as the outcome variable, a circular heat map was generated to visualise the findings of the Mendelian randomisation analysis performed on two sample groups. The heat map illustrated the causal effects estimated by five different Mendelian randomisation methods.

A causal relationship was found between one genus of gut microbiota and the risk of JIAU using the IVW method (**Figure 5**). MR analysis showed that genus *Prevotella7* reduced the likelihood of JIAU (OR: 0.398, 95% CI: 0.191–0.83, *p* = 0.014). Additionally, the MR-Egger, weighted median, simple mode and weighted mode methods revealed consistent effect directions. A circular heat map provided insights into the relationship between gut microbiota abundance (exposure variable) and JIAU (outcome variable) (**Figure 6**), with scatter plots (**Supplementary Figures 5**) and forest plots (**Supplementary Figure 6**) further illustrating these relationships.

### Reverse MR

For JIA, 13 SNPs and for JIAU, 6 SNPs met the IV screening criteria, each with an *F*-statistics > 10, indicating minimal instrumental bias (**Supplementary Tables 13**, **19**). Additionally, Steiger filtering analysis revealed no SNPs with opposing causal orientations for either condition (**Supplementary Tables 14**, **20**).

The SNP findings summarised data from a total of 208 gut microbiota classifications, including 16 classes, 126 genera, 33 families, 20 orders and 9 phyla, used to study the connection between JIA and gut microbiota (**Supplementary Table 15**). Significance thresholds for multiple comparisons at distinct taxonomic classifications were set as follows: phylum (*p* = 5.56×10^-3^), class (*p* = 3.13×10^-3^), order (*p* = 2.50×10^-3^), family (*p* = 1.52×10^-3^) and genus (*p* = 3.97×10^-4^), and were adjusted using the Bonferroni correction method. Similarly, for JIAU and gut microbiota, 208 different gut microbiota classifications were included in the data summary for the SNP results (**Supplementary Table 21**). These classifications encompassed 16 classes, 128 genera, 34 families, 20 orders and 9 phyla. Significance thresholds for multiple comparisons at different taxonomic classifications were set as follows: phylum (*p* = 5.56×10^-3^), class (*p* = 3.13×10^-3^), order (*p* = 2.50×10^-3^), family (*p* = 1.47×10^-3^) and genus (*p* = 3.91×10^-4^), and were adjusted using the Bonferroni correction method.

**Figure 7:**
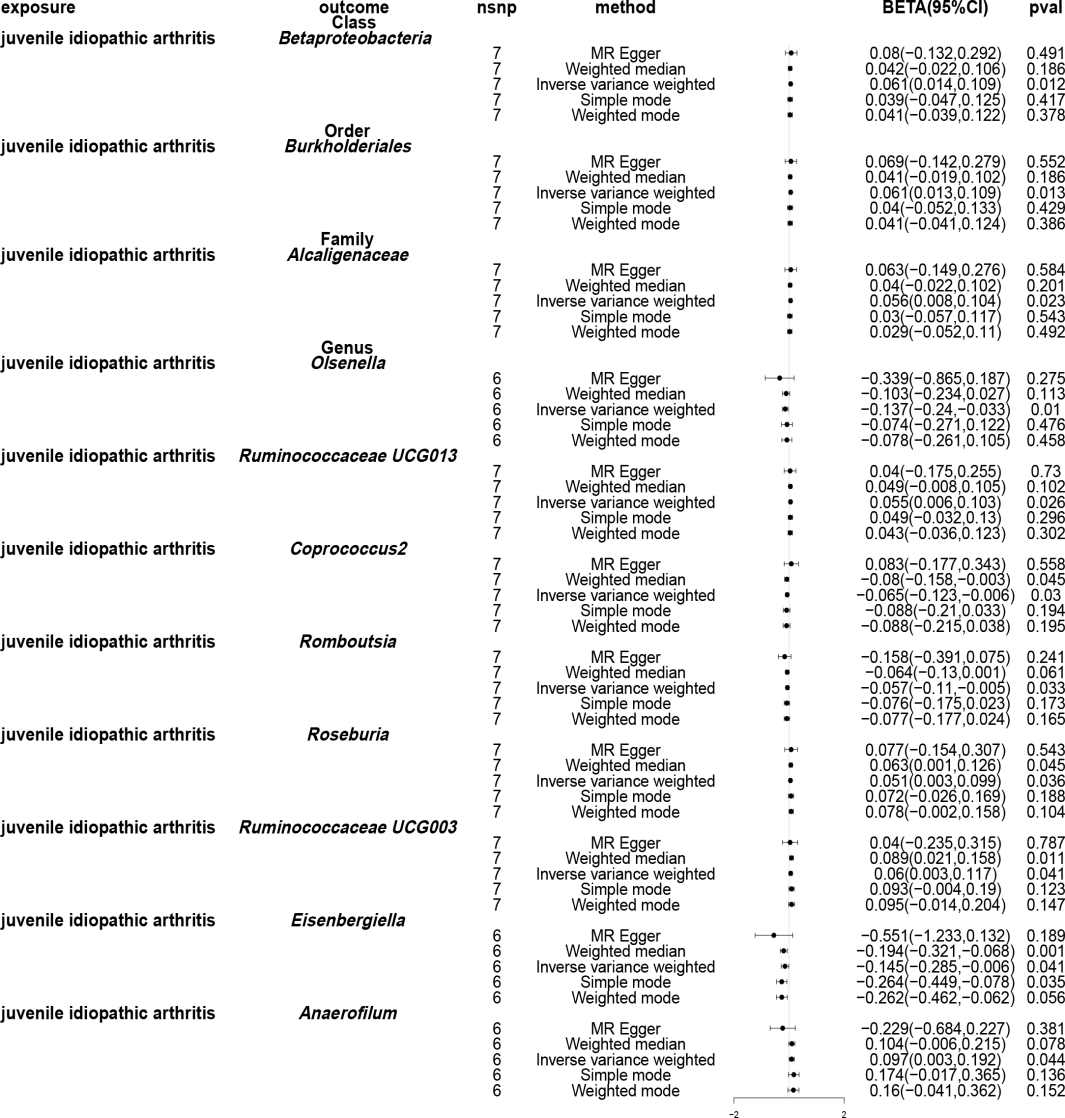
Forest plots of Mendelian randomisation for two samples, depicted the causal effects of five different Mendelian randomisation methods with juvenile idiopathic arthritis (JIA) as exposure and gut microbiota as the outcome. The effect estimates are presented as the effect size (BETA) and 95% confidence interval (CI). snp, single nucleotide polymorphism.

**Figure 8:**
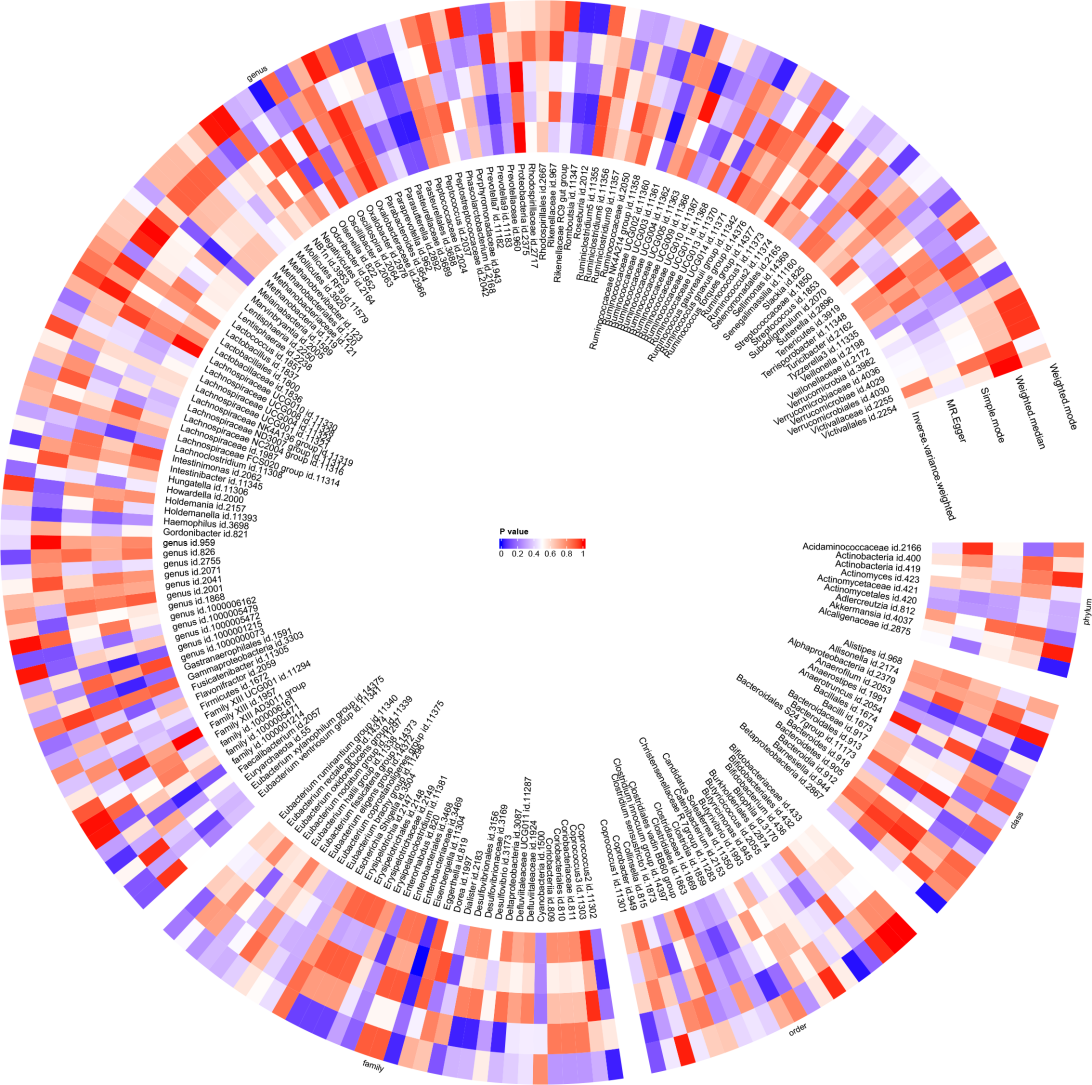
Circular heat map was generated to visually represent the outcomes of Mendelian randomisation analysis performed on two sample groups, depicting the causal effects estimated by five different Mendelian randomisation methods, with juvenile idiopathic arthritis (JIA) as the exposure variable and gut microbiota abundance as the outcome variable.

Using the IVW method, causal associations between JIA and gut microbiota were identified at one phylum, one order, one family and eight genera (**Figure 7**). MR analysis revealed that JIA increased the abundance of class *betaproteobacteria* (*β*: 0.061, 95%CI: 0.014–0.109, *p* = 0.012), order *Burkholderiales* (*β*: 0.061, 95%CI: 0.013–0.109, *p* = 0.013), family *Alcaligenaceae* (*β*: 0.056, 95%CI: 0.008–0.104, *p* = 0.023), genus *Ruminococcaceae UCG013* (*β*: 0.055, 95%CI: 0.006–0.103, *p* = 0.026), genus *Roseburia* (*β*: 0.051, 95%CI: 0.003–0.099, *p* = 0.036), genus *Ruminococcaceae UCG003* (*β*: 0.06, 95%CI: 0.003–0.117, *p* = 0.041) and genus *Anaerofilum* (*β*: 0.097, 95%CI: 0.003–0.192, *p* = 0.044), while decreasing the abundance of genus *Olsenella* (*β*: -0.137, 95%CI: -0.24- -0.033, *p* = 0.01), genus *Coprococcus2* (*β*: -0.065, 95%CI: -0.123- -0.006, *p* = 0.03), genus *Romboutsia* (*β*: -0.057, 95%CI: -0.11, -0.005, *p* = 0.033) and genus *Eisenbergiella* (*β*: -0.145, 95%CI: -0.285- -0.006, *p* = 0.041). For class *beta proteobacteria*, order *Burkholderiales*, family *Alcaligenaceae*, genus *Ruminococcaceae UCG013*, genus *Roseburia*, genus *Ruminococcaceae UCG003*, genus *Olsenella*, genus *Romboutsia* and genus *Eisenbergiella*, the MR-Egger, weighted median, mode-based estimator and weighted mode methods revealed directional effects that were consistent with the IVW method. However, for genus *Anaerofilum* and genus *Coprococcus2*, the results from the MR-Egger method differed from those of the IVW method, while the results from the weighted median, mode-based estimator and weighted mode methods aligned with the IVW method. A circular heat map visually represented the outcomes of MR analysis performed on the two sample groups, depicting the causal effects estimated by the five different MR methods and providing insights into the relationship between JIA (exposure variable) and gut microbiota abundance (outcome variable) (**Figure 8**). Scatter plots (**Supplementary Figure 9**) and forest plots **Supplementary Figure 10**) further illustrated these relationships.

**Figure 9:**
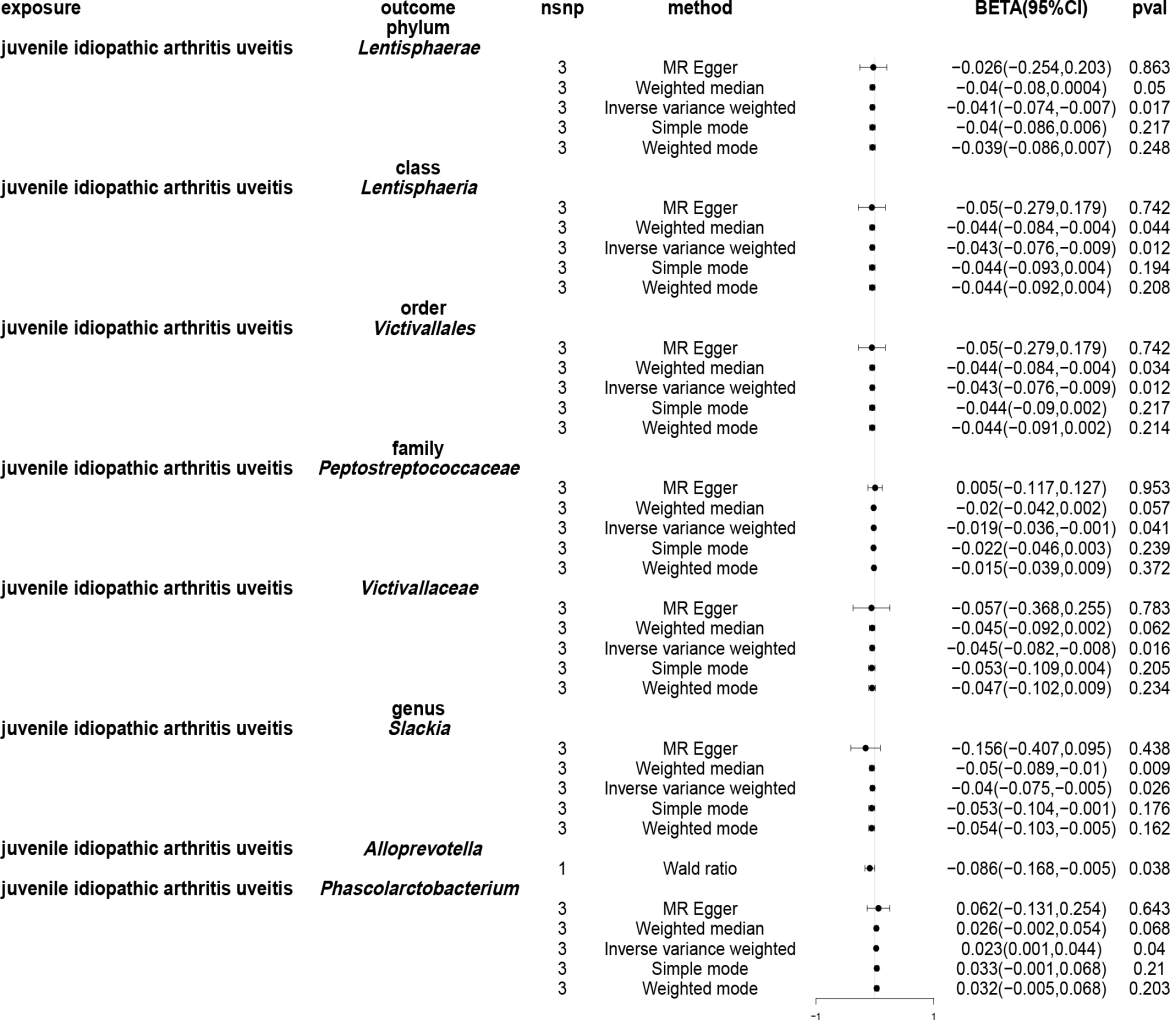
Forest plots of Mendelian randomisation for two samples illustrated the causal effects of five different Mendelian randomisation methods with juvenile idiopathic arthritis associated uveitis (JIAU) as exposure and gut microbiota as the outcome. The effect estimates are presented as the effect size (BETA) and 95% confidence interval (CI). snp, single nucleotide polymorphism.

**Figure 10:**
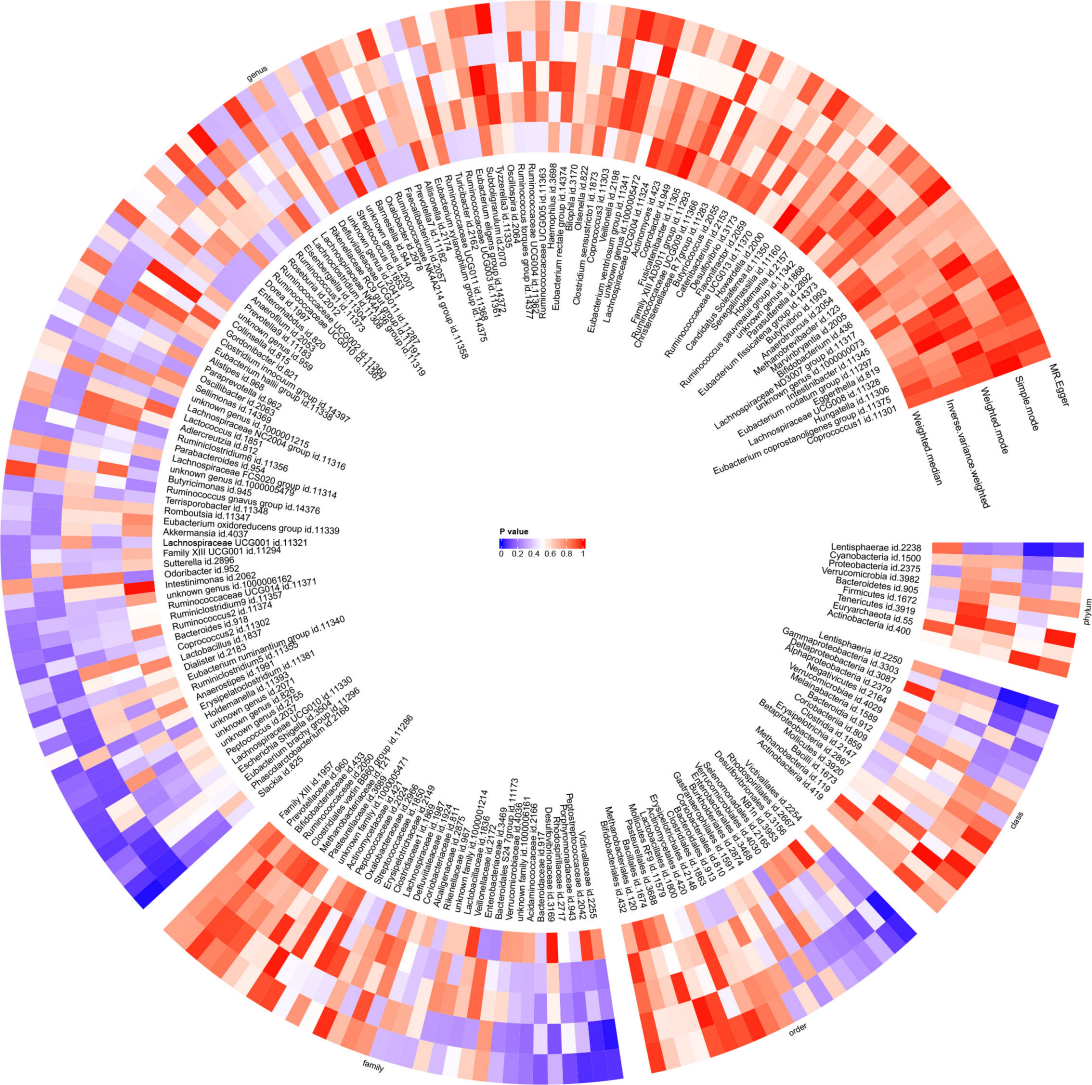
The results of Mendelian randomisation study on two sample groups were visualised via a circular heat map, which showed the causal effects estimated by five different Mendelian randomisation methods, with juvenile idiopathic arthritis associated uveitis (JIAU) as the variable of exposure and gut microbiota abundance as the variable of outcome.

Employing the IVW approach, a causal association was found between JIAU and the gut microbiota at one phylum, one class, one order, two families and three genera (**Figure 9**). MR analysis demonstrated that JIAU increased the abundance of genus *Phascolarctobacterium* (*β*: 0.023, 95%CI: 0.001–0.044, *p* = 0.04) but decreased the abundance of phylum *Lentisphaerae* (*β*: -0.041, 95%CI: -0.074–0.007, *p* = 0.017), class *Lentisphaeria* (*β*: -0.043, 95%CI: -0.076- -0.009, p=0.012), order *Victivallales* (*β*: -0.043, 95%CI: -0.076- -0.009, p=0.012), family *Peptostreptococcaceae* (*β*: -0.019, 95%CI: -0.036- -0.001, *p* = 0.041), family *Victivallaceae* (*β*: -0.045, 95%CI: -0.082- -0.008, *p* = 0.016), genus *Slackia* (*β*: -0.04, 95%CI: -0.075- -0.005, *p* = 0.026) and genus *Alloprevotella* (*β*: -0.086, 95%CI: -0.168- -0.005, *p* = 0.038). For phylum *Lentisphaerae*, class *Lentisphaeria*, order *Victivallales*, family *Victivallaceae*, genus *Slackia* and genus *Phascolarctobacterium*, MR-Egger, weighted median, simple mode and weighted mode methods revealed effect directions consistent with the IVW results. However, for the family *Peptostreptococcaceae*, the results from the MR-Egger method differed from that of the IVW method; however, the weighted median, simple mode and weighted mode methods revealed results consistent with the IVW findings. A circular heat map was generated to visually represent the outcomes of MR analysis performed on the two sample groups, depicting the causal effects estimated by the five different MR methods, thereby providing insights into the relationship between the exposure (JIAU) and outcome (gut microbiota abundance) (**Figure 10**). **Supplementary Figures 13**, **14** present the scatter and forest plots, respectively. **Supplementary Table 25** lists the top five microbial genera and species and their characterisation with potent effects associated with JIA or JIAU as derived from forward MR and reverse MR.

### Sensitivity analysis

When the variables of the gut microbiota or JIA or JIAU samples were analysed using the MR-Egger regression intercept method, no evidence of horizontal pleiotropy was detected (**Supplementary Tables 8**, **11**, **17**, **23**). We conducted outlier screening and removal in the MR-PRESSO analysis, and the global *p*-value test of MR-PRESSO did not indicate horizontal pleiotropy for gut microbiota or JIA or JIAU (**Supplementary Tables 9**, **12**, **18**). Furthermore, the majority of Cochrane’s Q test results did not reveal significant heterogeneity (*p* > 0.05) (**Supplementary Table 7**, **Supplementary Tables 10**, **16**, **22**). In the presence of significant heterogeneity, we employed random effects models and the IVW model as the main analytical results. Sensitivity analyses using the leave-one-out method and funnel plots are depicted in **Supplementary Figures 3**, **4**, **7**, **8**, **11**, **12**, **15**, **16**.

## Discussion

JIA is a common autoimmune rheumatic disease that poses a significant threat to the overall health and well-being of children, triggering joint deformities, functional impairments, stunted growth and osteoporosis, among other serious complications. Meanwhile, JIAU, a prevalent extra-articular complication of JIA, further complicates the clinical picture with chronic, non-granulomatous and non-infectious uveitis, often affecting the anterior segment of the eye. Approximately 10% to 14% of patients with JIA exhibit signs of uveitis before arthritis onset (41). Current treatment modalities for JIA and JIAU primarily focus on symptomatic relief, lacking definitive curative options (8). Previous studies have reported that siblings of patients with JIA have a higher risk of developing JIA than the general population, with the onset age, disease type and disease course of twin siblings exhibiting a similar trajectory (42). These findings indicate a genetic factor underlying JIA pathogenesis (43, 44). Moreover, subsequent studies have reported a genetic susceptibility locus for JIA, further confirming this speculation. However, as research advances, scientists have discovered that these genetic susceptibility factors can only explain 18% of the causes of JIA. Currently, the progression of JIA is speculated to be the outcome of the interaction between genetic susceptibility genes, environmental stimuli and immune dysregulation (45–47).

Emerging evidence underscores the microbiota’s involvement in immune-related disorders like RA and inflammatory bowel disease (IBD) (11). The intestinal microbiota predominately contributes to JIA pathogenesis by altering intestinal mucosal permeability and regulating the host immune system (48). Notably, altered microbial compositions, such as increased *phyla Bacteroidetes* and decreased *Firmicutes*, have been observed in paediatric patients with JIA compared to healthy individuals (15). This study suggests an increase in the abundance of class *betaproteobacteria* in patients with JIA, consistent with previous research findings (15).

In animal experiments, an extraperitoneal injection of intestinal bacterial cell wall components into mice has been demonstrated to induce arthritis in a conventional environment, but not in a sterile environment (49), suggesting a close relationship between gut microbiota alterations and arthritis development. Early investigations by Malin et al. (50) highlighted elevated bacterial urease activity in the stool samples of children with JIA compared to those of healthy children, suggesting an anaerobic dysbiosis of the intestinal flora. Interestingly, oral *Lactobacillus* administration to patients with JIA reduced urease activity. Results from a multicentre study conducted by van Dijkhuizen et al. (78 Italian children and 21 Dutch children) (17) revealed that Italian children with JIA exhibited an elevated abundance of *Faecalibacterium prausnitzii*, *Erysipelotrichaceae*, *Enterococcus*, *Parabactteroides* and *Ruminococccaceae*, but reduced levels of *Allobaculum*, *Gemellaceae*, *Propionibacterium acnes* and *Turicibacter* compared to the healthy control group. Similarly, our study also suggests an increase in the abundance of genera *Ruminococcaceae UCG013* and *Ruminococcaceae UCG003* in patients with JIA. Additionally, a significant decrease in the level of intestinal flora has been reported in samples from active and inactive states compared to healthy children (17). Kindgren et al.’s study on population queues (51) revealed a higher content of *Acidaminococcales*, *Prevotella 9* and *Veillonella parvula* in JIA cases, while *Coprococcus, Subdoligranulum, Phascolarctobacterium, Dialister spp, Bifidobacterium breve, Fusicatenibacter saccharivorans, Roseburia intestinalis* and *Akkermansia muciniphila* exhibited reduced abundance. Notably, studies have linked the presence of *Parabacteroides distasonis* to an increased risk of subsequent JIA occurrence, alongside shorter breastfeeding duration and increased antibiotic exposure, particularly in genetically susceptible populations. Therefore, it can be concluded that environmental stimuli have a stronger effect on genetically predisposed infants and that microbial dysbiosis during infancy may initiate or accelerate the development of JIA. However, t our MR study findings indicate an increase in the abundance of genus *Phascolarctobacterium* in JIAU, contrasting previous results. This inconsistency may stem from JIAU’s nature as a complication of JIA, potentially exerting different effects on the gut microbiota. However, these findings support the notion that environmental stimuli exert a significant influence. In another cross-sectional investigation, Qian et al. reported that the JIA group exhibited a significant reduction in the relative abundance of four genera (*Anaerostipes*, *didiister*, *Lachnospira* and *Roseburia*) compared to the control group (18). These four genera are known to produce short-chain fatty acids (SCFAs), whose decrease is associated with severe clinical complications. Additionally, the study identified the genus *Lachnospiraceae UCG001* as potentially reducing the risk of JIA. However, contrary to previous findings (18), an increase in the level of genus *Roseburia* in JIA was observed in our study.

Previous research consistently highlights differences in the gut microbiota composition between children with JIA and their healthy counterparts. However, the causal relationship between gut microbiota and the disease remains unclear. It is uncertain whether microbiota imbalance precedes JIA or arises as a consequence of long-term inflammation, abnormal metabolism or behavioural changes associated with JIA symptoms. Given the limitations of previous studies, including susceptibility to confounding factors and reverse causation effects, further investigation is warranted to elucidate the link between JIA or JIAU and the gut microbiota. In our study, leveraging GWAS data and MR analysis, we investigated the causality between JIA or JIAU and gut microbiota. In addition to our primary findings, we observed an elevated risk of *FamilyXI*, while genera *Rikenellaceae RC9* gut group, *Intestinimonas* and *Clostridium* innocuum group were linked to decreased JIA risk. Conversely, a reduced abundance of genus *Prevotella7* was associated with a decreased likelihood of JIAU. Furthermore, the abundance of order *Burkholderiales*, family *Alcaligenaceae* and genus *Anaerofilum* increased, while the abundance of genera *Olsenella*, *Coprococcus2*, *Romboutsia* and *Eisenbergiella* decreased in JIA cases. JIAU decreased the abundance of phylum *Lentisphaerae*, class *Lentisphaeria*, order *Victivallales*, family *Peptostreptococcaceae*, family *Victivallaceae* and genus *Slackia*. These novel findings contribute to existing knowledge by uncovering previously unexplored associations.

Given the pivotal role of gut microbiota in arthritis, targeted probiotics are emerging as a novel therapeutic avenue for rheumatic diseases. Since the mature and stable state of the microbiota once formed is difficult to change, and childhood is a critical period to acquire basic functions (such as immune tolerance to commensal microbiota), these findings present a unique opportunity for early intervention and potentially modifying the disease progression by targeting the microbiota (12). Clinical trials investigating prebiotics and probiotics are on the rise, with an expanding body of evidence reporting favourable tolerance and potential benefits for restoring infant microbiota to health (52, 53). However, a randomised controlled trial of probiotics by Shukl et al. demonstrated good tolerance in patients with Enthesitis-related arthritis (ERA) but did not show any favourable clinical or immunological effects compared to non-steroidal anti-inflammatory medication therapy (54). Therefore, to assess the efficacy and safety of probiotics for JIA, further clinical data are warranted to ensure better recommendations for clinical practice.

Our research offers several advantages. To the best of our knowledge, this is the first investigation to explore the causal relationship between JIA/JIAU and gut microbiota abundance using MR analyses. Relative to other research methods, MR analyses have several advantages in the study of the causal relationship between gut microbiota and JIA or JIAU. First, in terms of evidence-based medicine levels of evidence, MR has the third highest evidence-based rating after systematic reviews of randomised controlled trials (RCTs) and RCTs when RCTs are feasible, and the highest evidence-based rating when RCTs are not feasible, ranking first (55). Whereas, due to practical and ethical reasons, random assignment of specific gut flora cannot be done, RCT studies are not feasible when conducting causal studies of gut flora with JIA and JIAU. Observational study data are relatively more readily available and closer to real-world situations, but problems such as smaller sample sizes, confounding factors, and causal inversions often limit inferences about cause and effect (56), so appropriate causal modelling is needed to infer causal associations between exposure factors and disease outcomes. Animal studies have an even lower evidence-based rating. Therefore, for the study of the causal relationship of gut microbiota with JIA or JIAU, MR studies provide an effective way to solve the above problems. By utilising MR, which relies on the random allocation of allelic genes at conception, we established a temporal relationship (‘cause before effect’) between genetic variations and disease development, which is free from the influence of postnatal environmental factors and social behaviours. This approach minimised the risk of reverse causal linkages and allowed for more effective control of confounding variables. Moreover, to ensure the robustness of the auxiliary variables used in the MR analysis, we sourced GWAS data for gut microbiota, JIA and JIAU from the largest available database. Furthermore, to minimise the possible effects of weak IV bias, we adopted appropriate thresholds for the genomic instruments based on a threshold of *p*< 1.0×10^-5^ in this bidirectional study. This criterion was selected based on the availability of an adequate number of SNPs with suitable statistical power for the majority of gut microbiota to effectively prevent confounding.

While our study boasts several strengths, it also has certain limitations. Firstly, although our analysis yields statistically significant *p*-values, they may not be as robust as those corrected using the Bonferroni method for significance. Nevertheless, our research is hypothesis-driven and supported by substantial biological data and past studies that establish the epidemiological link between gut microbiota and JIA or JIAU. However, future research may need to include samples from a larger population of patients with JIA and JIAU to further strengthen these results. Secondly, adjusting *p*-values for multiple comparisons may increase the risk of false positives and potentially weaken the number and multi-level structure of microbial communities (abundance and correlation between microbial strains) as well as the correlation between JIA or JIAU. Therefore, cautious interpretation is warranted when considering unfavourable outcomes or potentially significant *p*-values. Furthermore, the two-sample MR method is a theoretical causal analysis method, and the conclusions of our study may contradict epidemiological studies suggesting the impact of environmental risk factors on JIA or JIAU. Additionally, as the specific mechanisms of the gut microbiome in the onset of JIA or JIAU remain unclear, further research, including clinical studies and other ex vivo and *in vivo* studies such as animal experiments, is needed to verify the results of this study. New research techniques and methods such as metagenomics and metabolomics technologies, where available, are needed to further validate the results of the present study and to understand the relationship between the observed intestinal flora and JIA, as well as the mechanisms behind the relationship of JIAU. Lastly, as our study primarily includes participants of European descent in the JIA group, generalising our results to other ethnic populations should be done with caution. When GWAS summary data on JIA, JIAU and gut microbiota from other races become available in the future, we also hope to analyse and study them to improve the applicability and generalisability of these findings.

## Conclusion

This study presents compelling evidence supporting a causal association between genetically predicted gut microbiota and JIA or JIAU development. This study underscores the significant interactive influence of intestinal flora on these conditions, suggesting their potential as novel biomarkers for diagnosis and prevention. These findings offer valuable insights that can aid in addressing and mitigating the impact of JIA or JIAU.

## Data availability statement

The original contributions presented in the study are included in the article/**Supplementary Material**. Further inquiries can be directed to the corresponding authors.

## Author contributions

J-BH: Conceptualization, Writing – original draft, Writing – review & editing, Formal analysis, Methodology, Validation, Visualization. Y-XC: Formal analysis, Writing – original draft, Writing – review & editing, Methodology, Validation, Visualization. Z-YS: Formal analysis, Validation, Writing – original draft, Writing – review & editing. X-YC: Methodology, Validation, Writing – review & editing. Y-NL: Conceptualization, Validation, Writing – review & editing. J-HY: Conceptualization, Funding acquisition, Writing – review & editing, Validation.
