# Supplementary material for: Causal association of juvenile idiopathic arthritis or JIA-associated uveitis and gut microbiota: a bidirectional two-sample Mendelian randomisation study
Source: Front Immunol. 2024 Jul 24;15:1356414. doi: 10.3389/fimmu.2024.1356414 (PMC11303189; doi:10.3389/fimmu.2024.1356414)
Supplement: Supplementary file 1 [file DataSheet_1.docx]

**
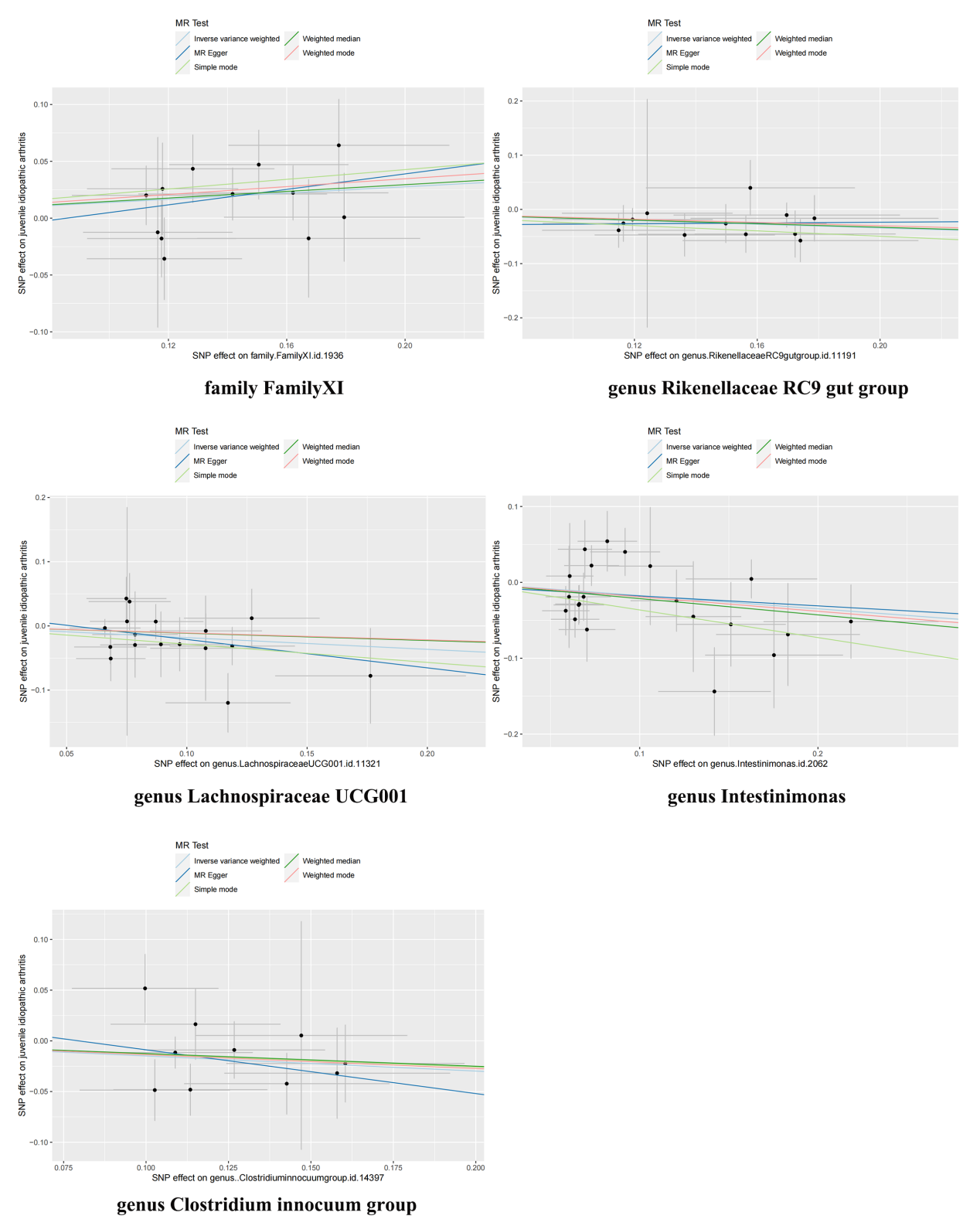
**

**Figure S1** Scatter plot of results of Mendelian randomization with gut microbiota abundance as exposure and JIA as outcome. (Only results with significant *p*-values in the IVW method are shown)


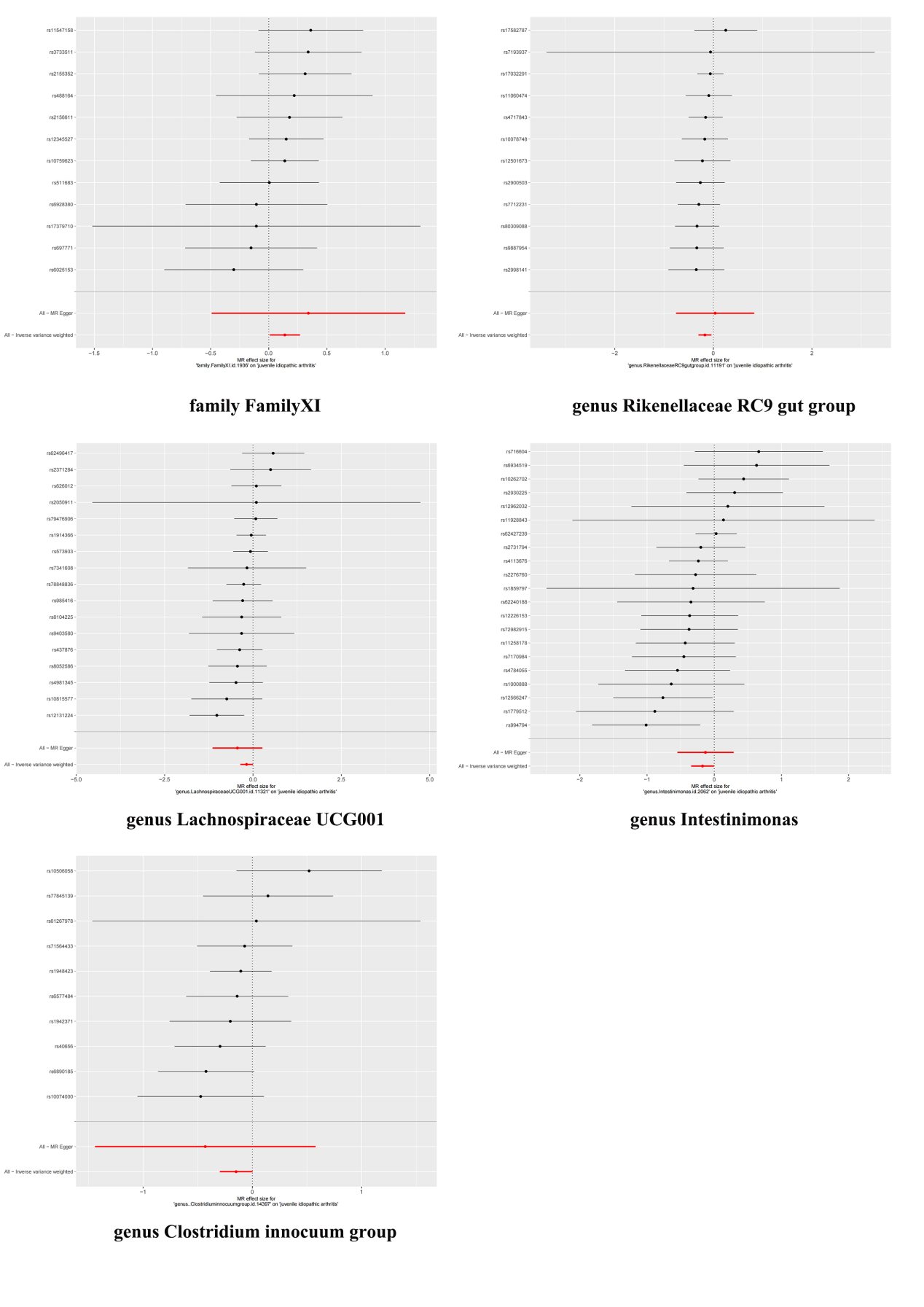


**Figure S2** Forest plot of single SNP analysis using Wald ratios, with gut microbiota abundance as exposure and JIA as outcome. (Only results with significant *p*-values in the IVW method are shown)


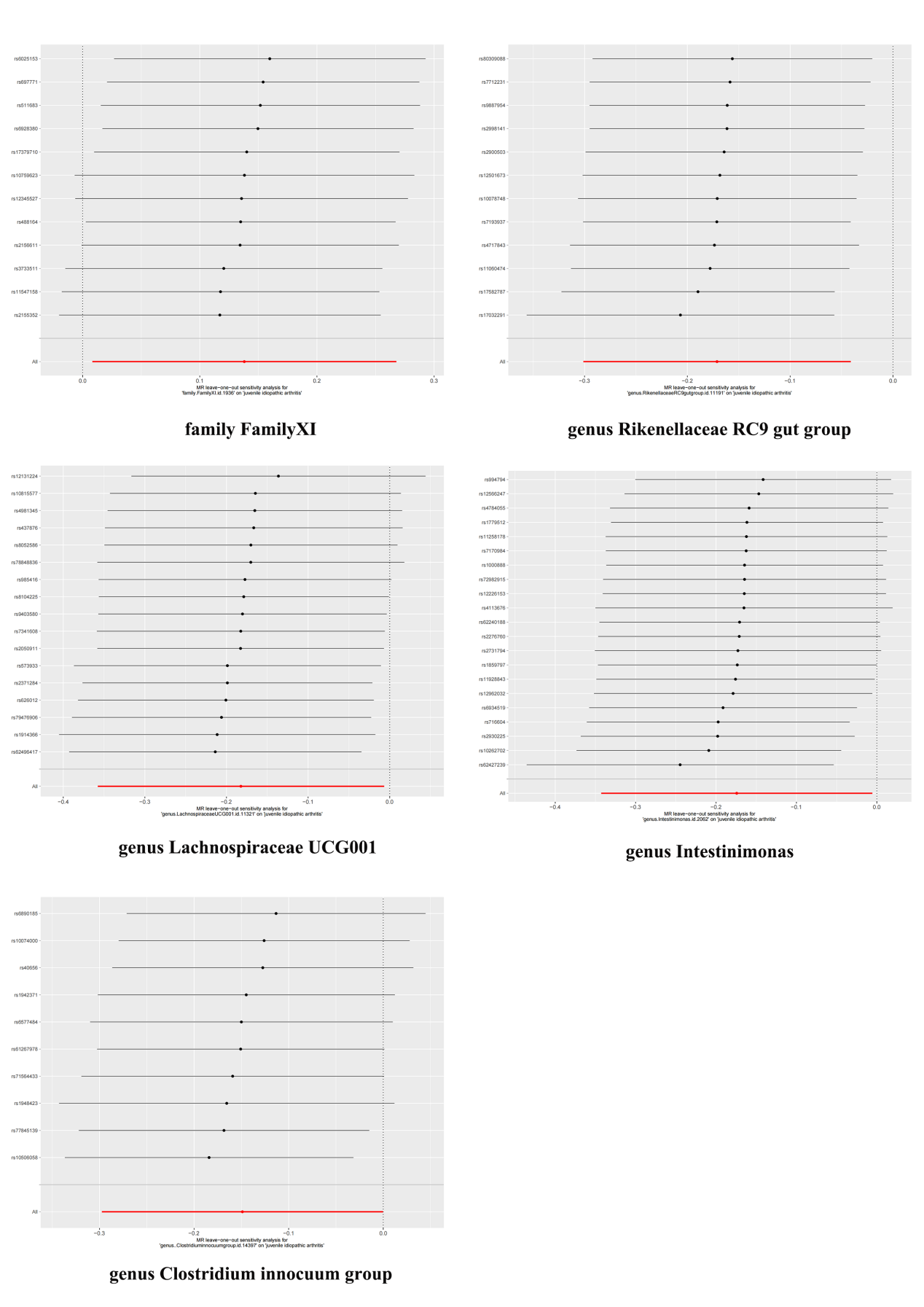


**Figure S3** Leave-one-out analysis plot, with gut microbiota abundance as exposure and JIA as result. (Only results with significant *p*-values in the IVW method are shown)


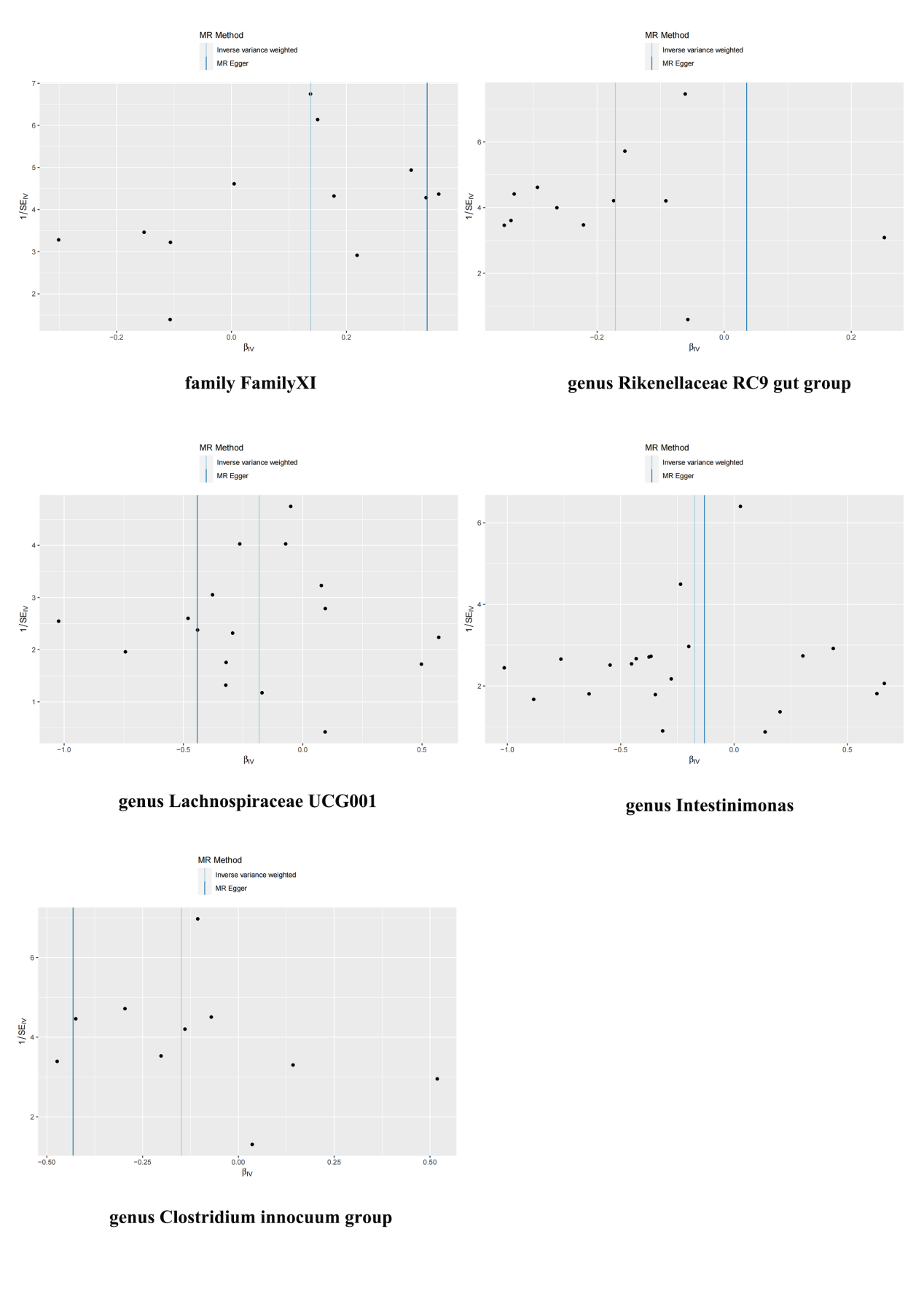


**Figure S4** Funnel plot, with gut microbiota abundance as exposure and JIA as result. (Only results with significant *p*-values in the IVW method are shown)


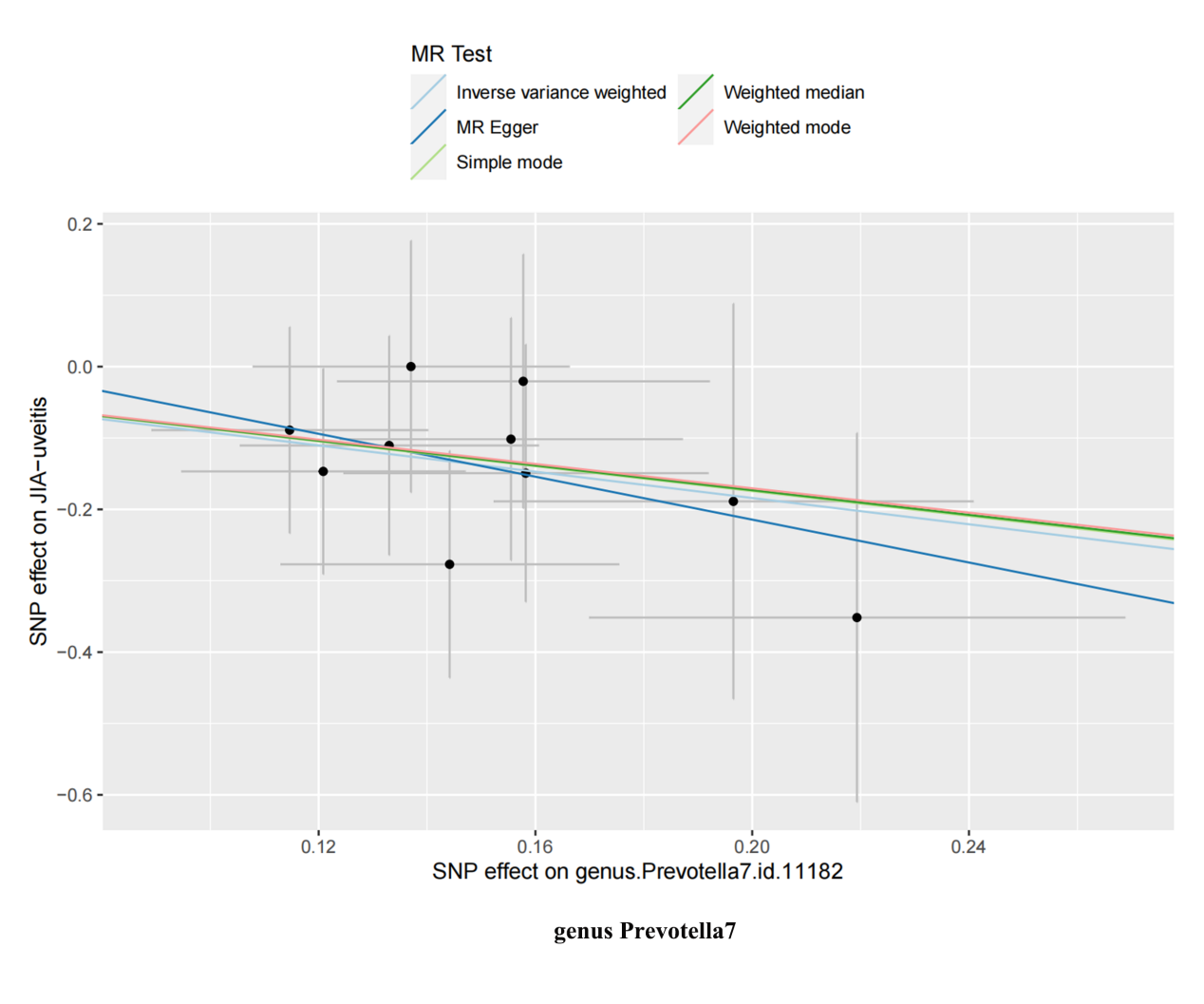


**Figure S5** Scatter plot of results of Mendelian randomization with gut microbiota abundance as exposure and JIAU as outcome. (Only results with significant *p*-values in the IVW method are shown)


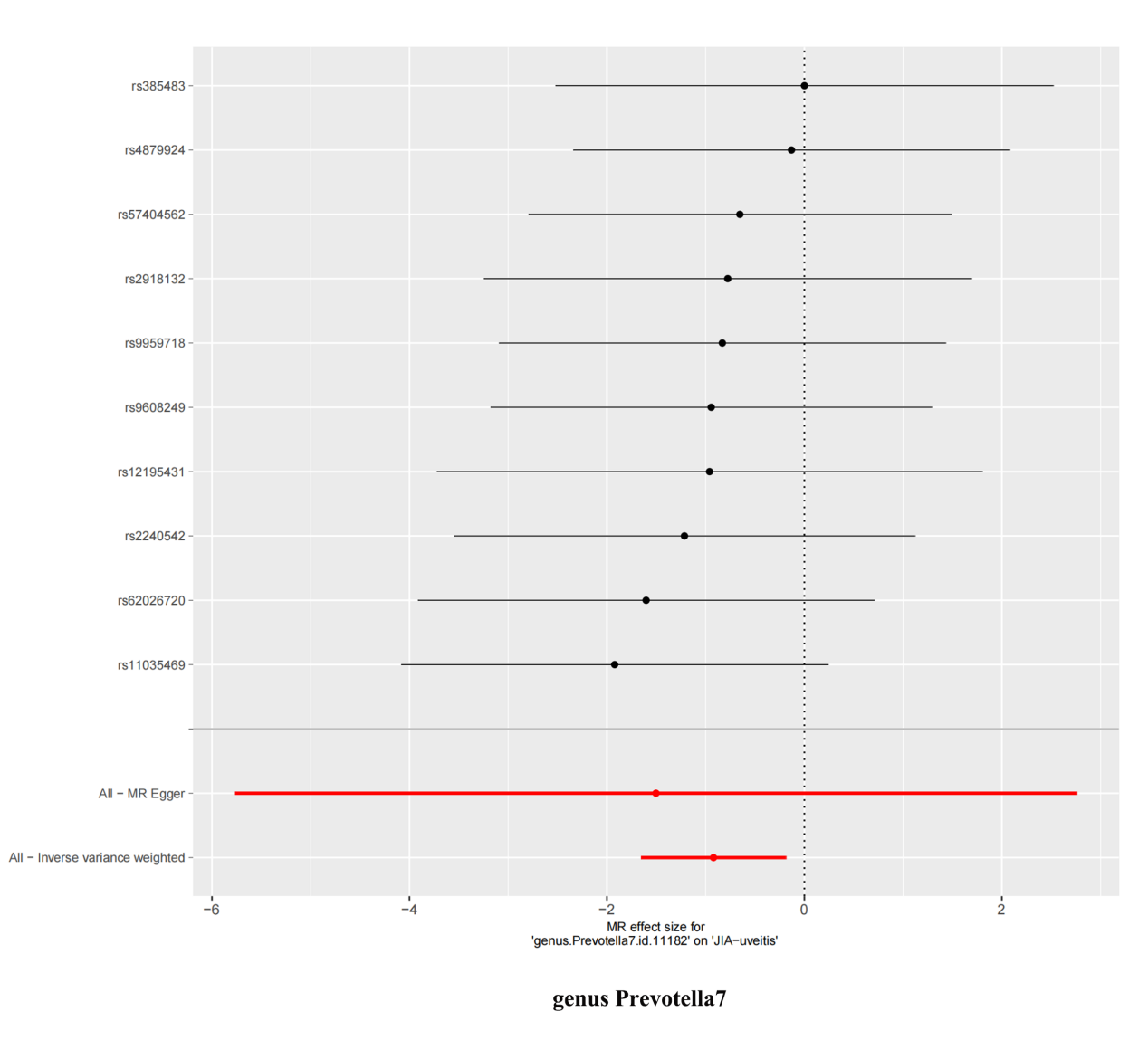


**Figure S6** Forest plot of single SNP analysis using Wald ratios, with gut microbiota abundance as exposure and JIAU as outcome. (Only results with significant *p*-values in the IVW method are shown)


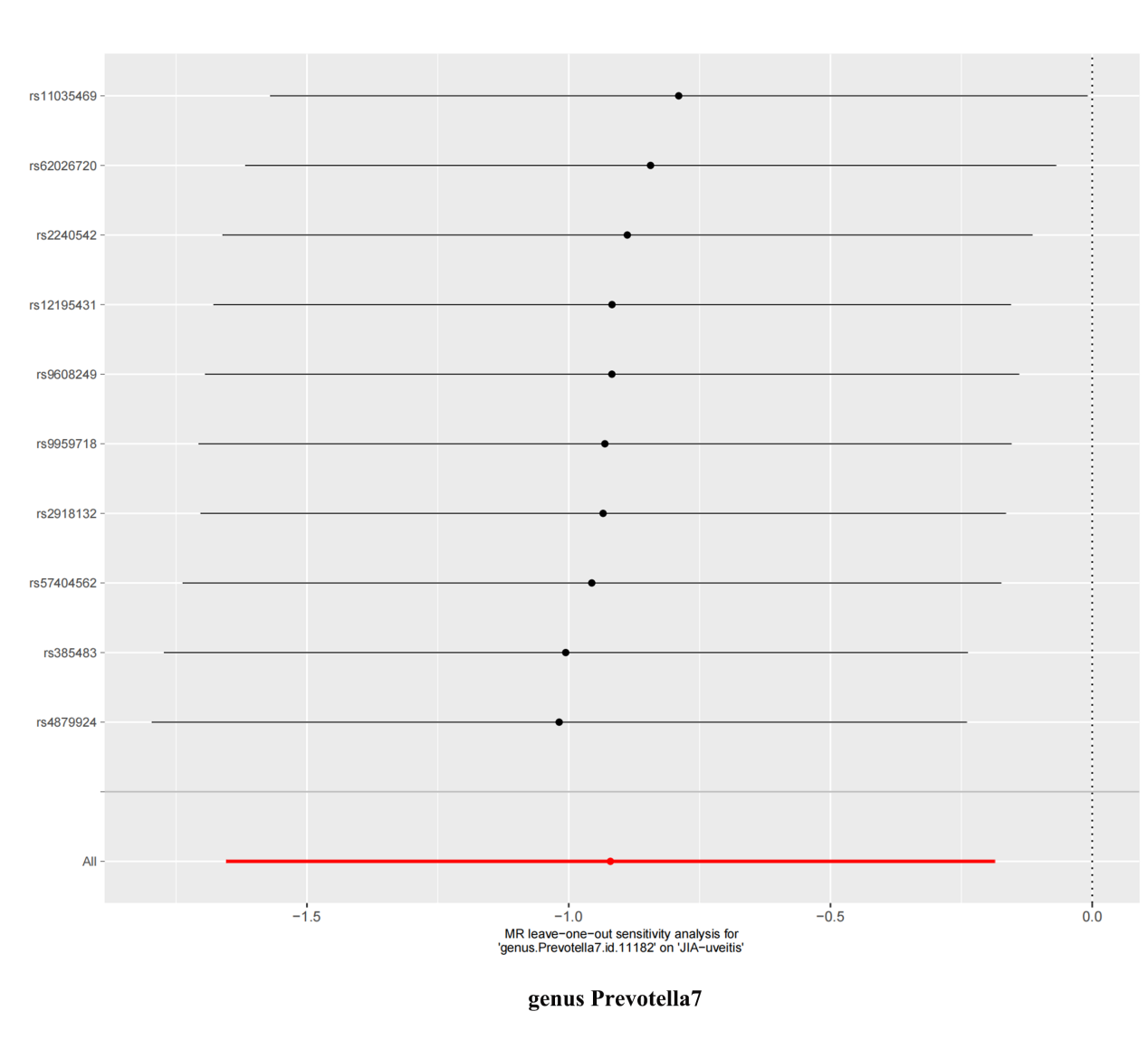


**Figure S7** Leave-one-out analysis plot, with gut microbiota abundance as exposure and JIAU as result. (Only results with significant *p*-values in the IVW method are shown)


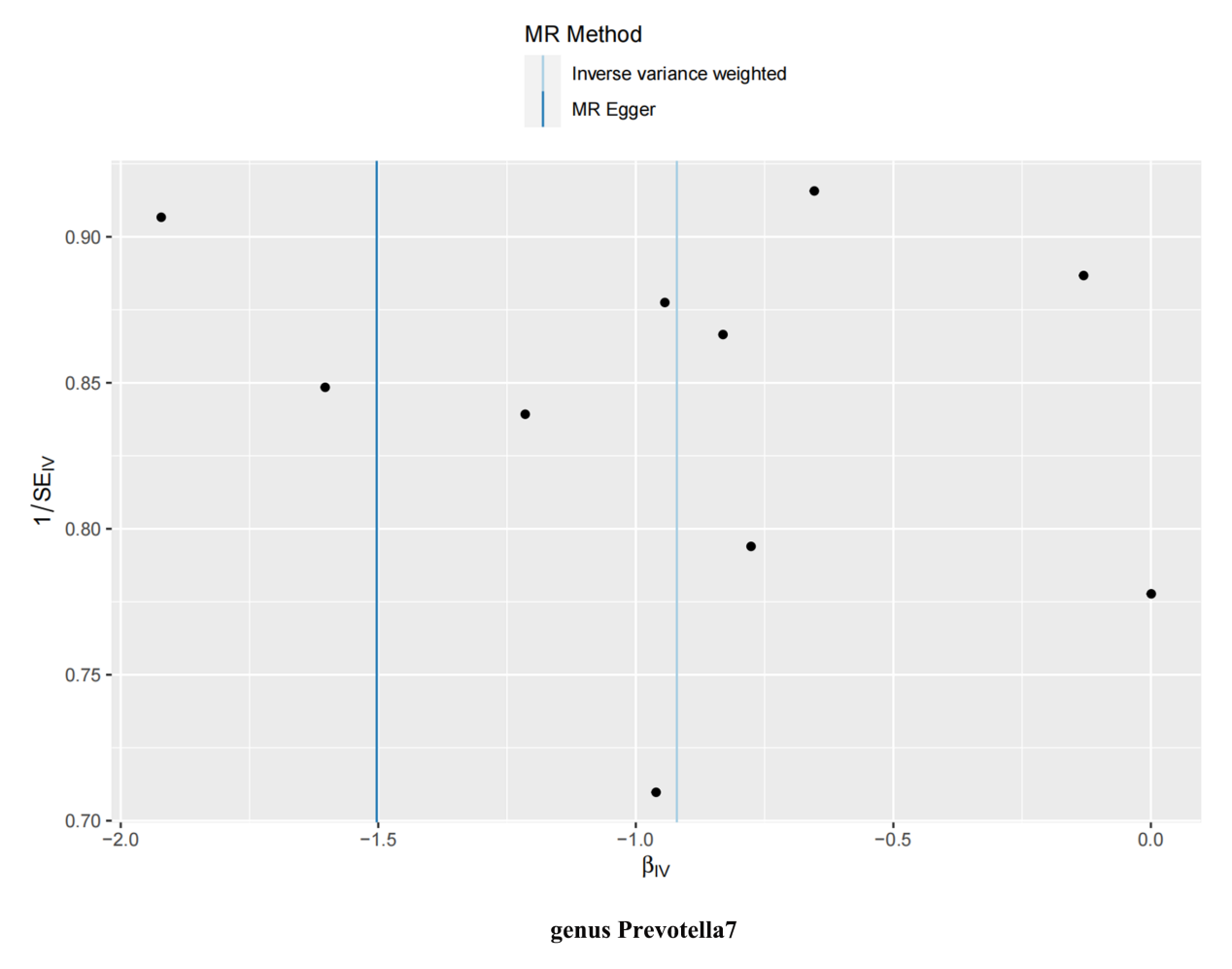


**Figure S8** Funnel plot, with gut microbiota abundance as exposure and JIAU as result. (Only results with significant *p*-values in the IVW method are shown)


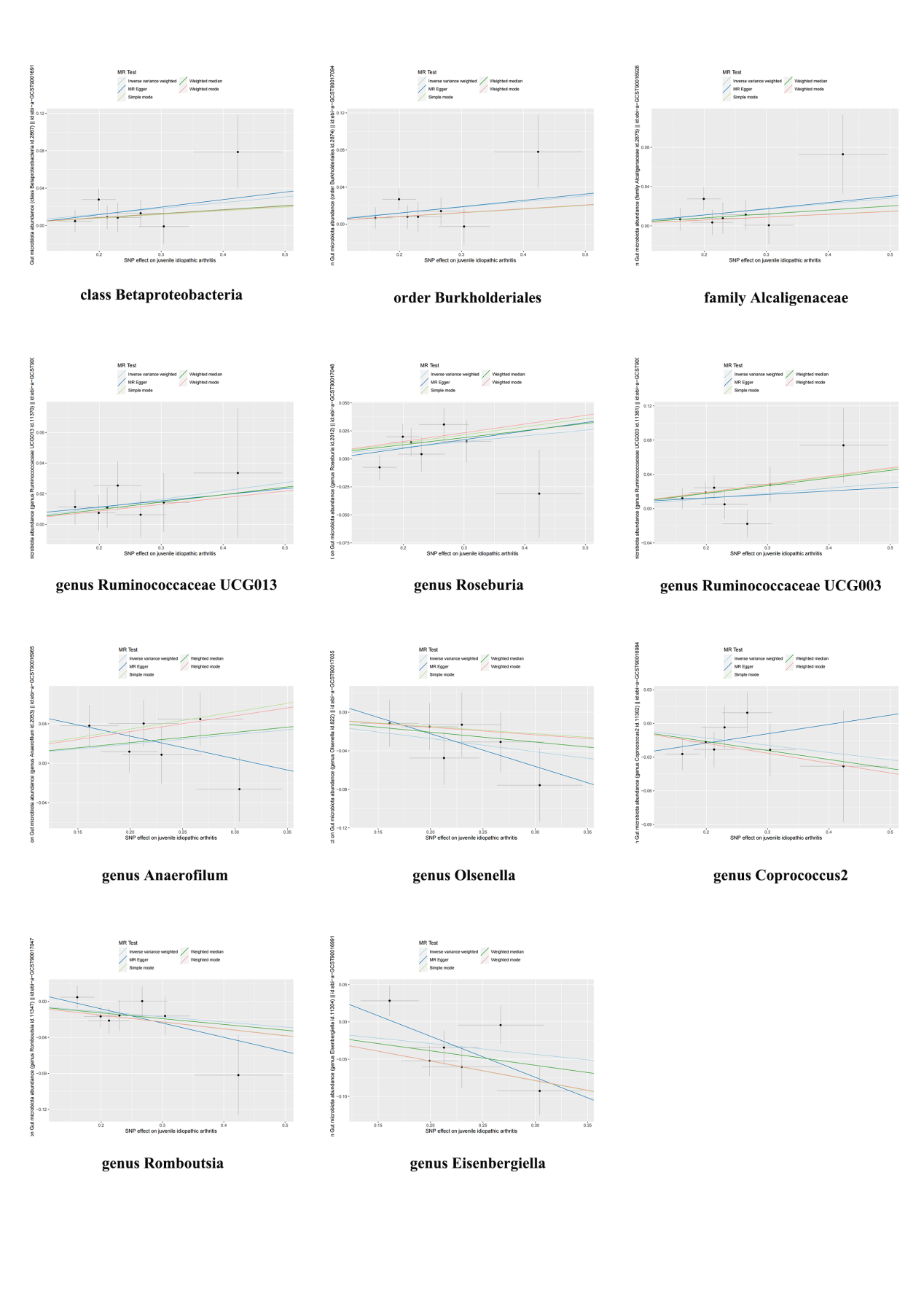


**Figure S9** Scatter plot of results of Mendelian randomization with JIA as exposure and gut microbiota abundance as outcome. (Only results with significant *p*-values in the IVW method are shown)


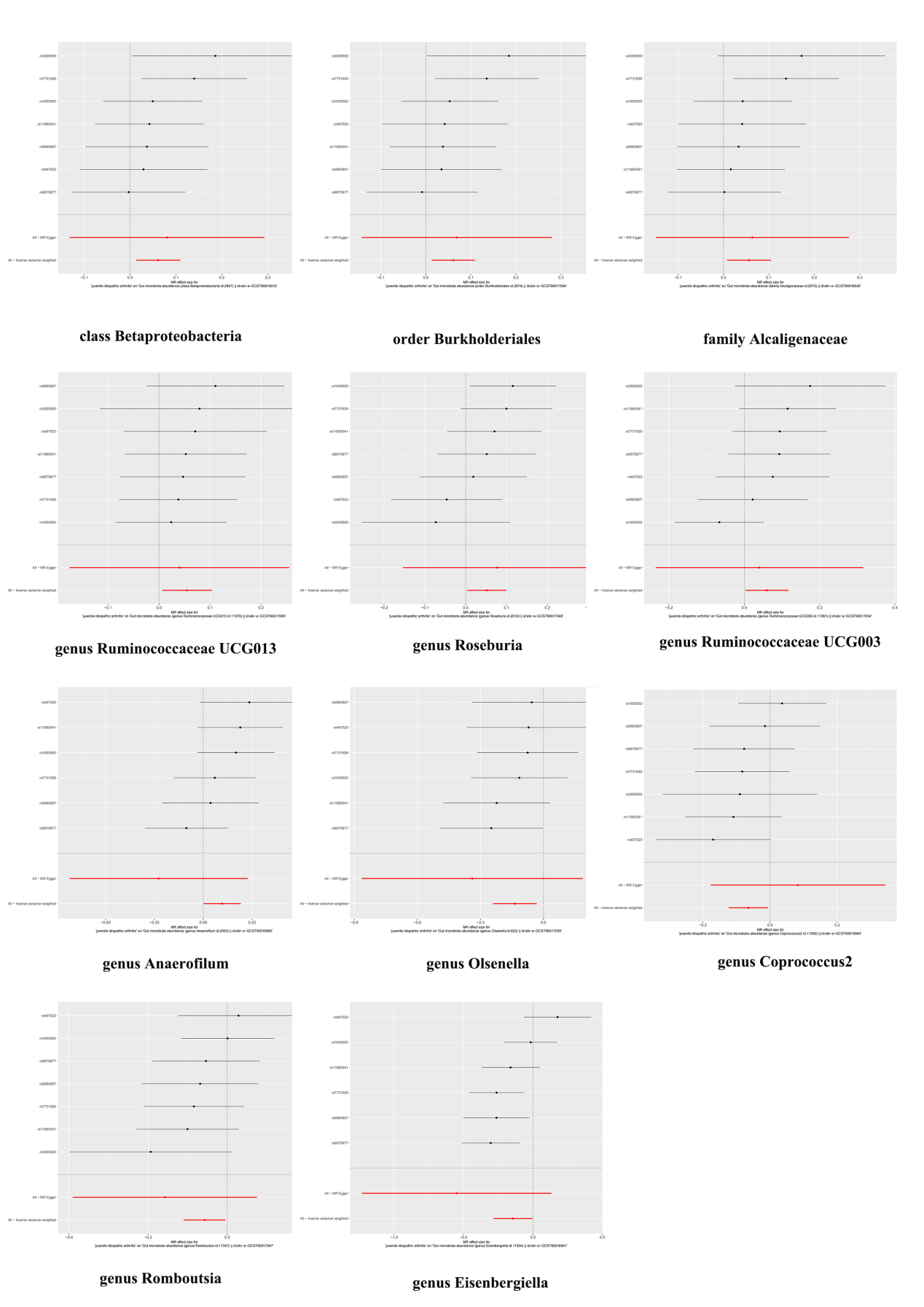


**Figure S10** Forest plot of single SNP analysis using Wald ratios, with JIA as exposure and gut microbiota abundance as outcome. (Only results with significant *p*-values in the IVW method are shown)


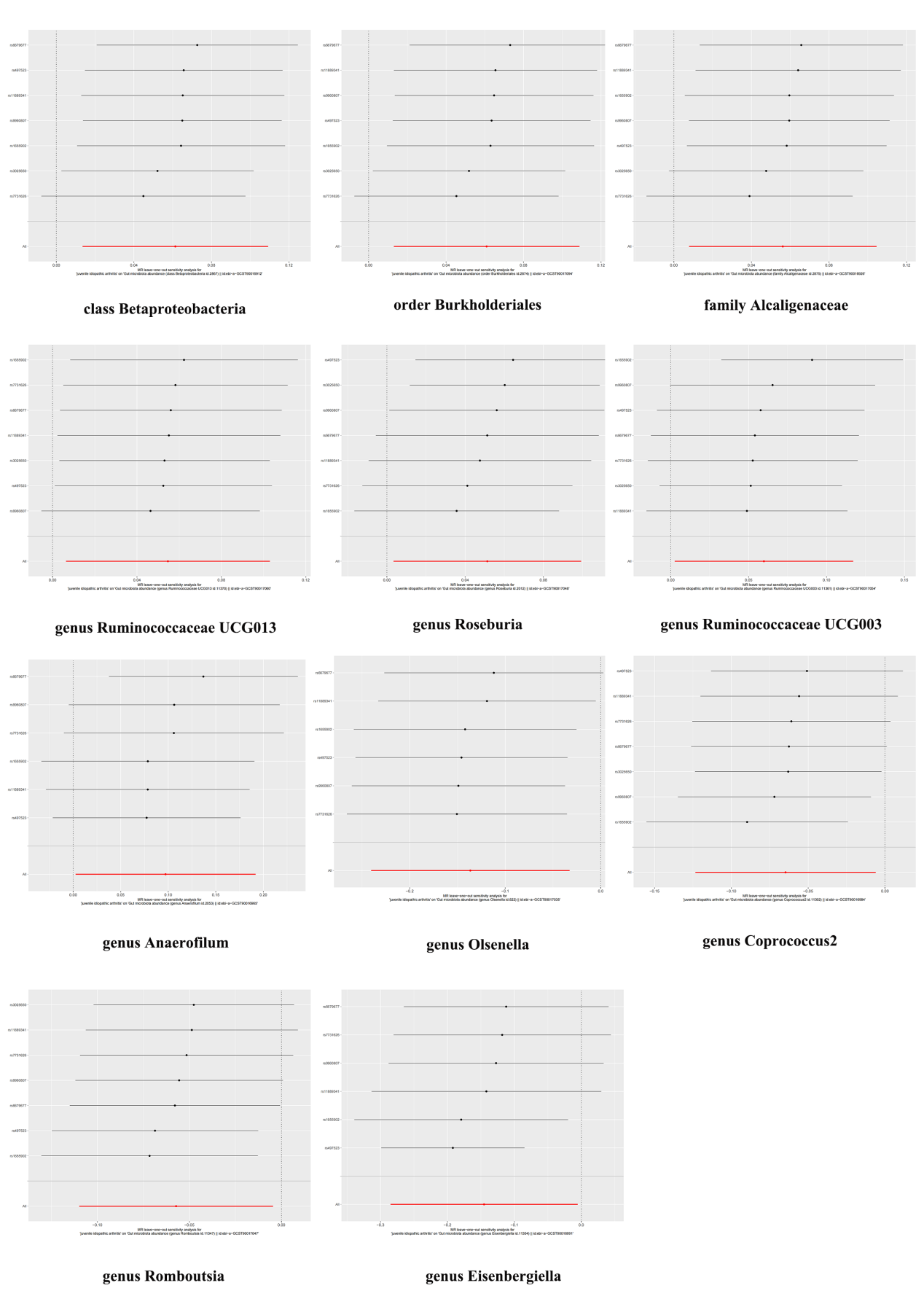


**Figure S11** Leave-one-out analysis plot, with JIA as exposure and gut microbiota abundance as result. (Only results with significant *p*-values in the IVW method are shown)


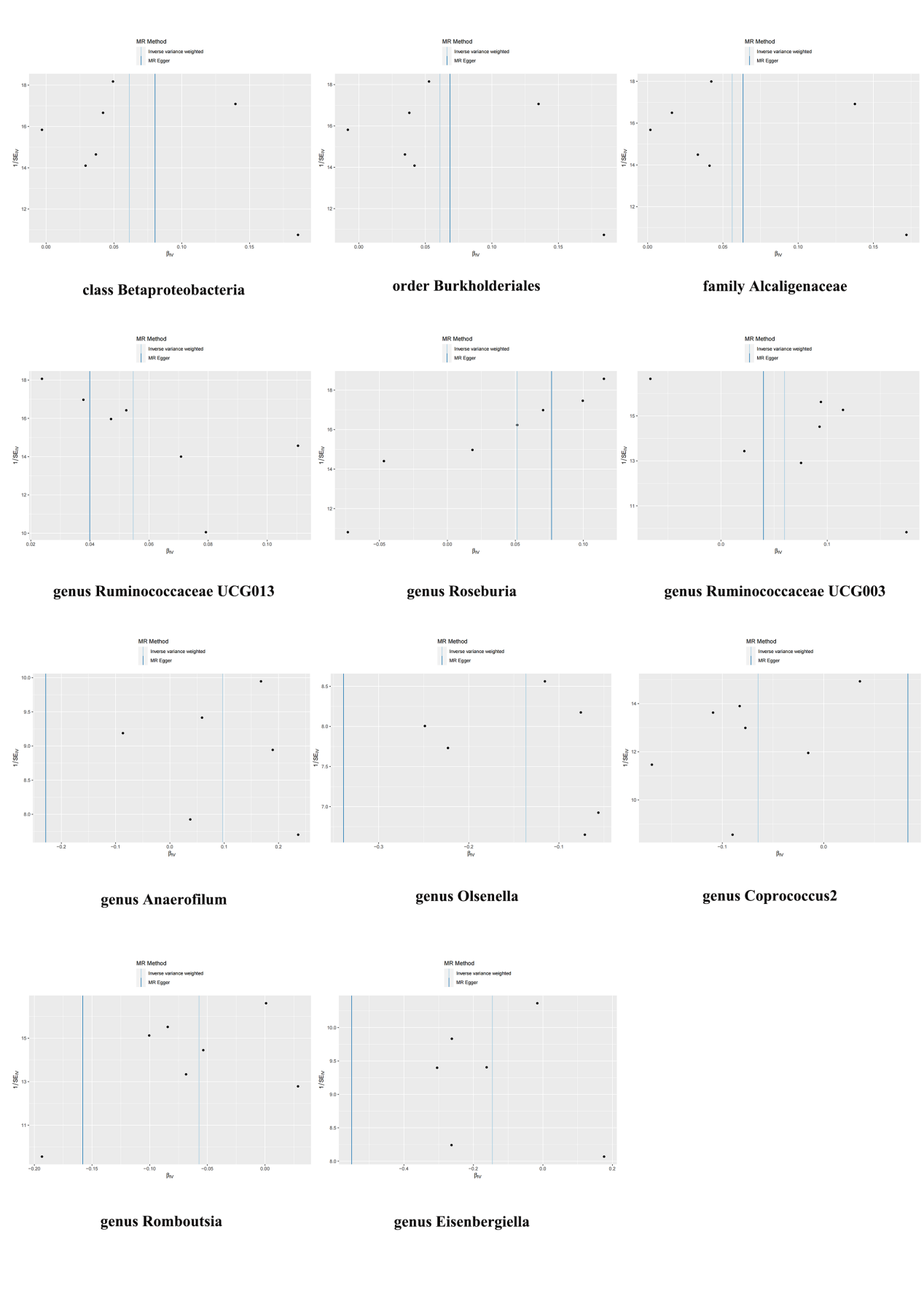


**Figure S12** Funnel plot, with JIA as exposure and gut microbiota abundance as result. (Only results with significant *p*-values in the IVW method are shown)


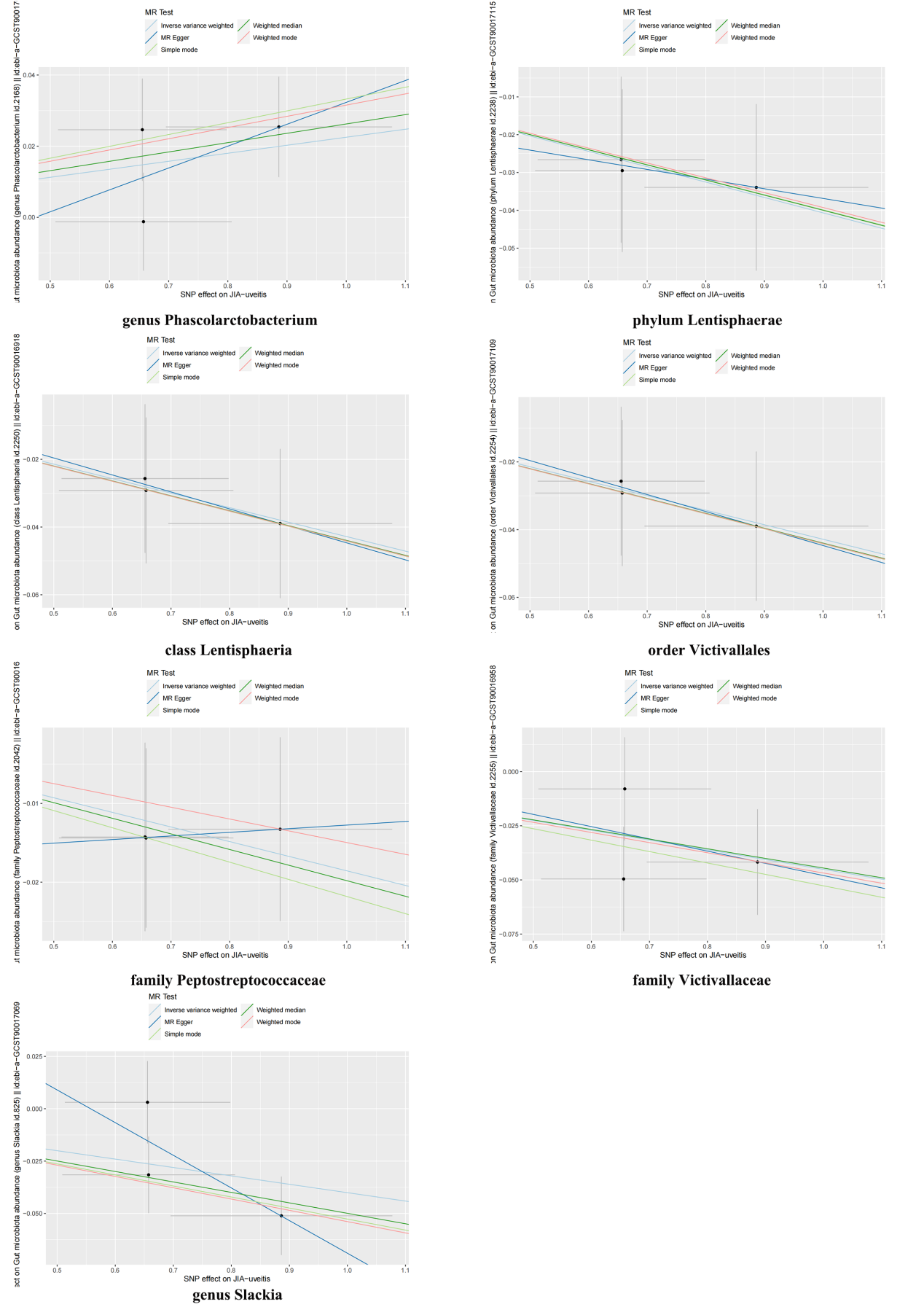


**Figure S13** Scatter plot of results of Mendelian randomization with JIAU as exposure and gut microbiota abundance as outcome. (Only results with significant *p*-values in the IVW method are shown)


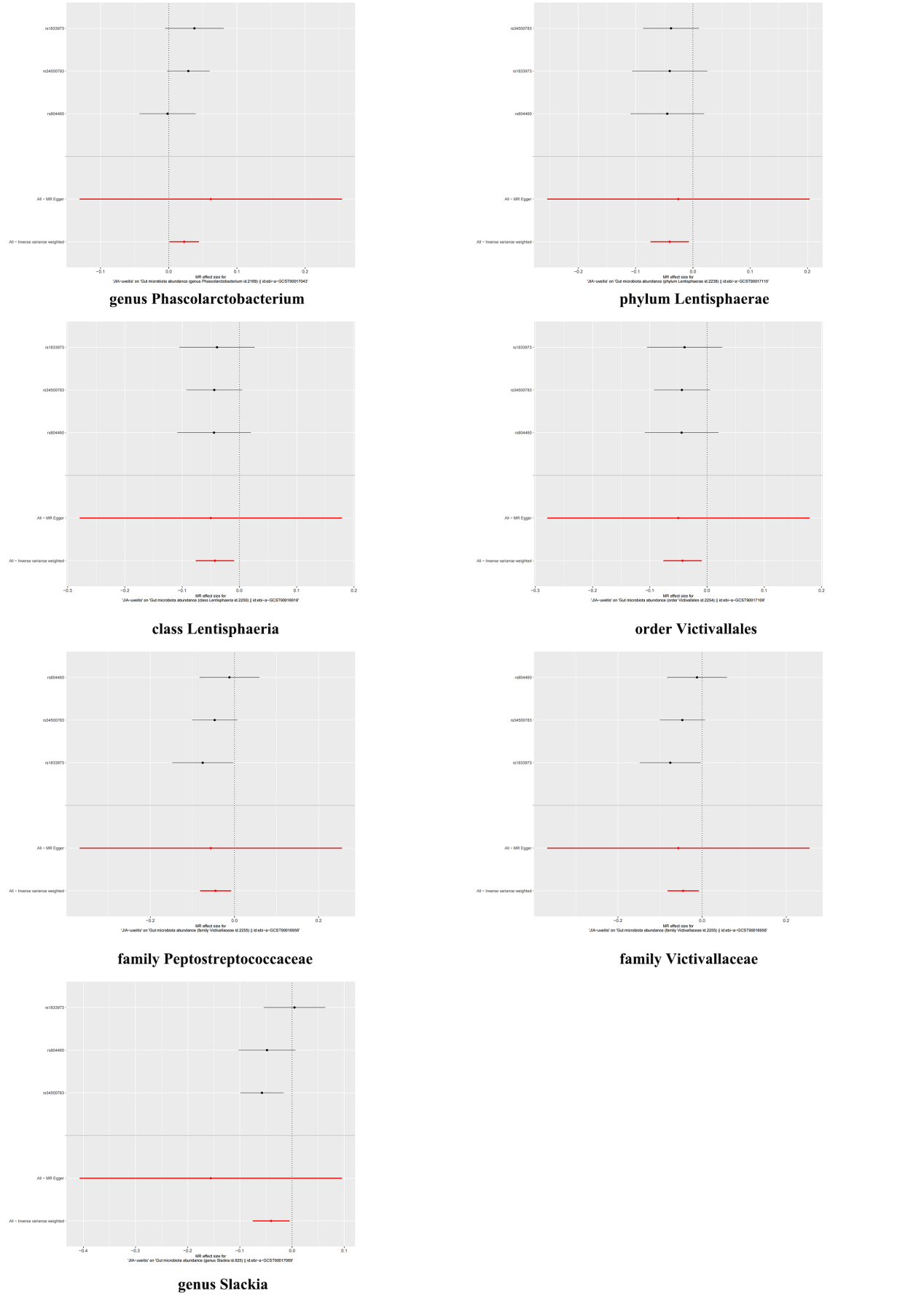


**Figure S14** Forest plot of single SNP analysis using Wald ratios, with JIAU as exposure and gut microbiota abundance as outcome. (Only results with significant *p*-values in the IVW method are shown)


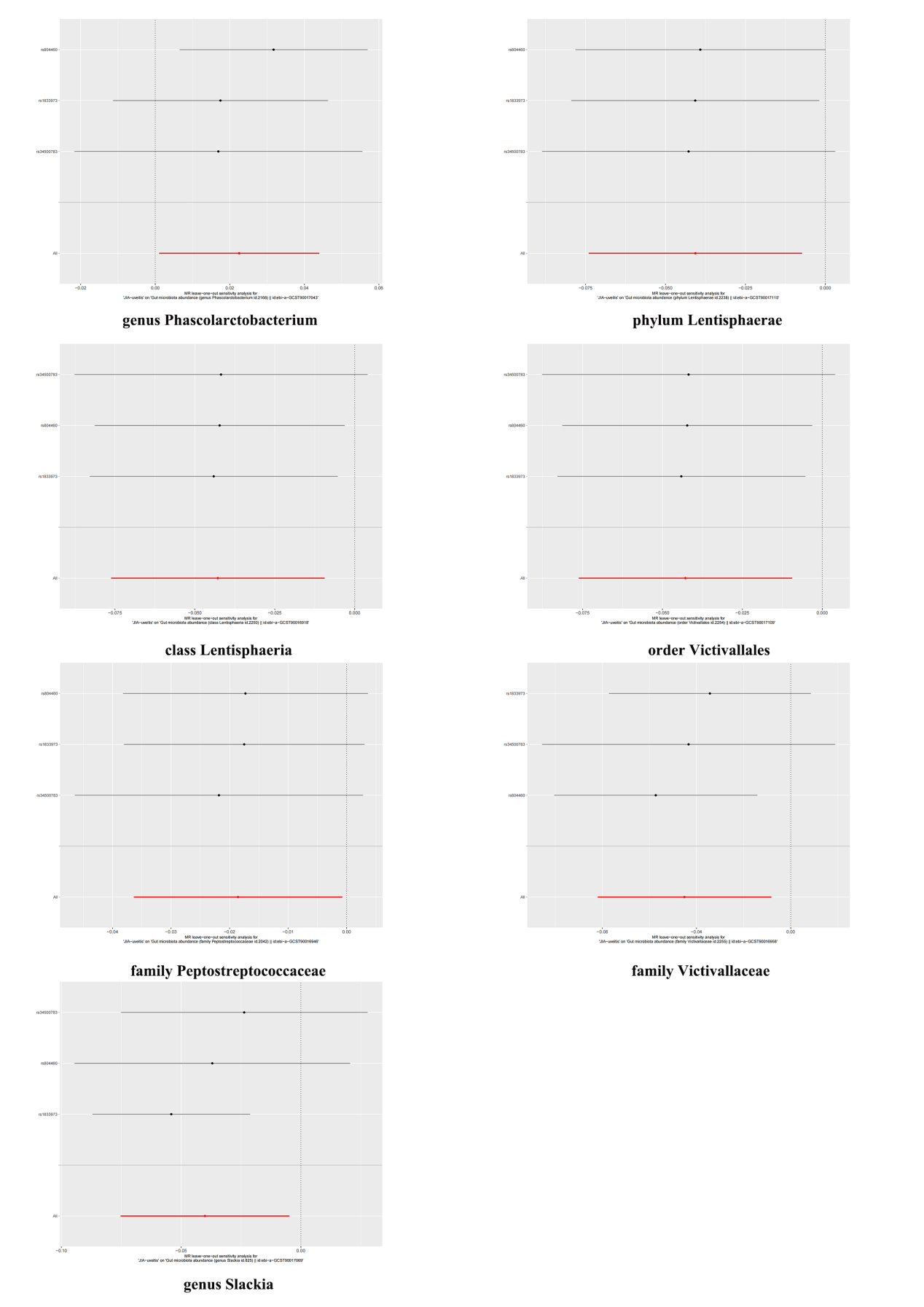


**Figure S15** Leave-one-out analysis plot, with JIAU as exposure and gut microbiota abundance as result. (Only results with significant *p*-values in the IVW method are shown)


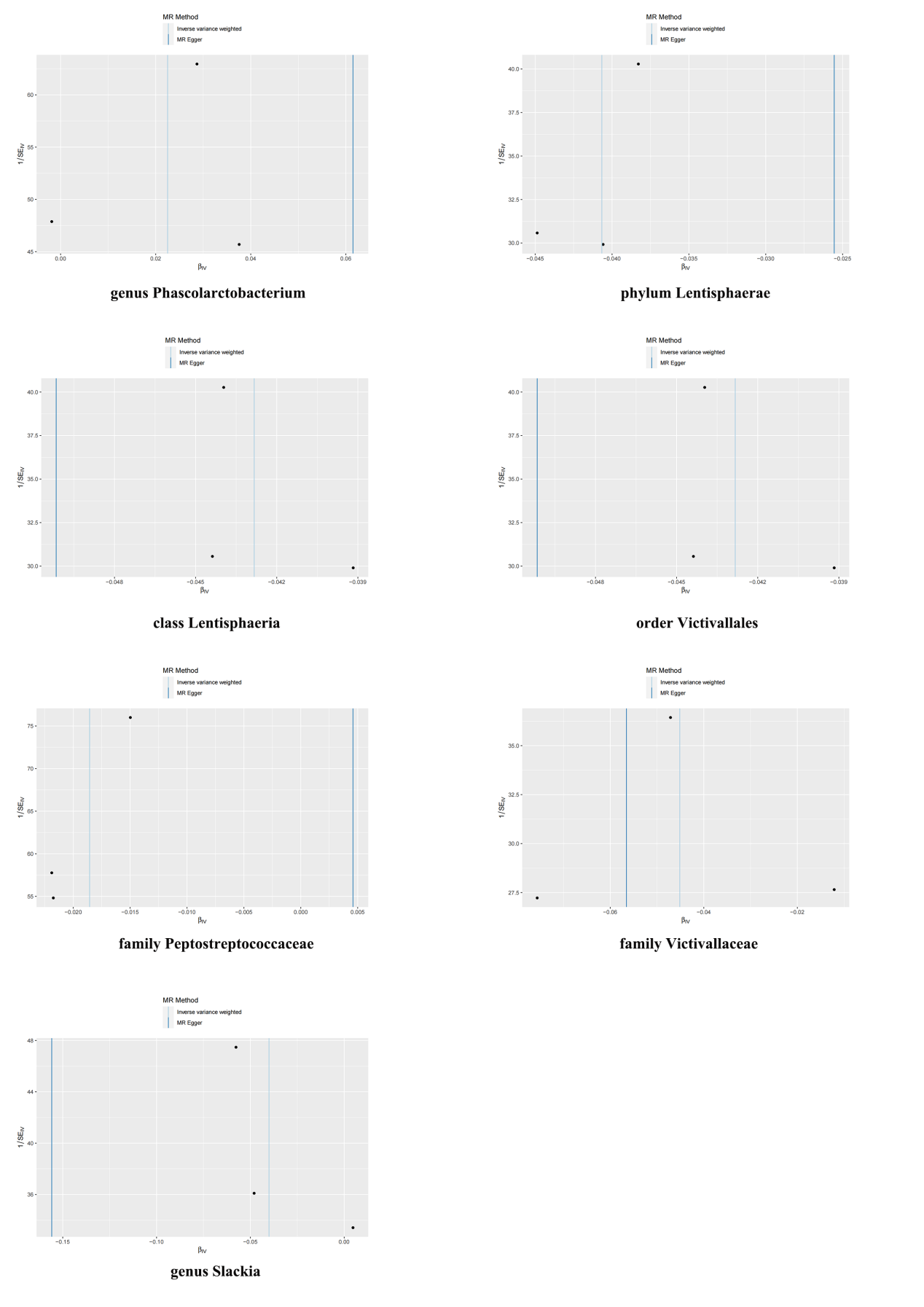


**Figure S16** Funnel plot, with JIAU as exposure and gut microbiota abundance as result. (Only results with significant *p*-values in the IVW method are shown)
