# Supplementary material for: Causal association of juvenile idiopathic arthritis or JIA-associated uveitis and gut microbiota: a bidirectional two-sample Mendelian randomisation study
Source: Front Immunol. 2024 Jul 24;15:1356414. doi: 10.3389/fimmu.2024.1356414 (PMC11303189; doi:10.3389/fimmu.2024.1356414)
Supplement: Supplementary file 2 [file DataSheet_2.docx]

**STROBE-MR checklist of recommended items to address in reports of Mendelian randomization studies**^1^ ^2^

| **Item No.** | **Section** | **Checklist item** | **Page No.** | **Relevant text from manuscript** |
| --- | --- | --- | --- | --- |
| 1 | **TITLE and ABSTRACT** | Indicate Mendelian randomization (MR) as the study’s design in the title and/or the abstract if that is a main purpose of the study | 1,2 | Title: Association of Juvenile Idiopathic Arthritis (JIA) and JIA-associated Uveitis and Gut Microbiota: A Bidirectional Two-sample Mendelian Randomization Study  Methods: A two-sample bidirectional Mendelian randomization (MR) study was conducted using the largest existing gut microbiota, juvenile idiopathic arthritis (JIA) and JIA-associated Uveitis (JIAU) genome-wide association studies (GWAS). |
|  | **INTRODUCTION** |  |  |  |
| 2 | **Background** | Explain the scientific background and rationale for the reported study. What is the exposure? Is a potential causal relationship between exposure and outcome plausible? Justify why MR is a helpful method to address the study question | 3,4 | Introduction: Juvenile idiopathic arthritis (JIA) is a heterogeneous disease characterized by arthritis of unknown origin that occurs before the age of 16, which can result in functional limitations and disability in severe cases. JIA-associated uveitis (JIAU) is commonly recognized as a prevalent and severe extra-articular manifestation of JIA . Recent evidence indicates that the gut microbiota may be a potential factor in immune-mediated diseases, such as rheumatoid arthritis (RA), that are linked to alterations in the gut microbiota. However, the causal relationship between JIA or JIAU and the gut microbiota remains uncertain,which poses obstacles to the prevention and treatment of JIA/JIAU. Furthermore, observational studies may be influenced by potential confounding factors and reverse causality, which can introduce biases in the results. Thus, it is imperative to study the causal link between the gut microbiota and JIA/JIAU. |
| 3 | **Objectives** | State specific objectives clearly, including pre-specified causal hypotheses (if any). State that MR is a method that, under specific assumptions, intends to estimate causal effects | 4 | Introduction: Building upon the recent large-scale genome-wide association studies (GWAS) on the gut microbiota and disease, we employed the Mendelian randomization approach to investigate the causal link between the gut microbiota and the risk of JIA/JIAU in this study. This study aims to explore the impact of genetic prediction of JIA/JIAU on the gut microbiota, and elucidate the role of the gut microbiota in the pathogenesis of JIA/JIAU through genetic prediction. |
|  | **METHODS** |  |  |  |
| 4 | **Study design and data sources** | Present key elements of the study design early in the article. Consider including a table listing sources of data for all phases of the study. For each data source contributing to the analysis, describe the following: |  | Table listing sources of data for all phases of the study: Table S1 |
|  | a) | Setting: Describe the study design and the underlying population, if possible. Describe the setting, locations, and relevant dates, including periods of recruitment, exposure, follow-up, and data collection, when available. | 4,5 | GWAS SUMMARY DATA FOR JIA  The GWAS data for JIA is derived from a research of 6,056 JIA patients and 25,086 European ancestry controls recently updated. The detailed research process is described in the studies by Lopez-Isac E and McIntosh LA.  GWAS SUMMARY DATA FOR JIAU  The GWAS data for JIAU is sourced from the study by Haasnoot et al. , which included 192 JIAU patients of European ancestry and 330 JIA control patients without uveitis.  GWAS SUMMARY DATA FOR GUT MICROBIOTA  Data on the composition of human gut microbiota were obtained from the MiBioGen consortium through a large-scale multi-ethnic GWAS study. This study involved 18,340 participants from 24 cohorts in countries such as the United States, Canada, Israel, Korea, Germany, Denmark, the Netherlands, Belgium, Sweden, Finland, and the UK. |
|  | b) | Participants: Give the eligibility criteria, and the sources and methods of selection of participants. Report the sample size, and whether any power or sample size calculations were carried out prior to the main analysis | 4,5 | GWAS SUMMARY DATA FOR JIA  A total of 4520 UK JIA samples and 9965 healthy individuals were recruited for the study. JIA DNA samples were genotyped on the Illumina Infinium CoreExome and Infinium OmniExpress genotyping arrays. Sample-level quality control (QC) was applied based on the following exclusion criteria: call rate <0.98 and discrepancy between genetically inferred sex and database records. SNPs that were non-autosomal, had a call rate <0.98 or a minor allele frequency (MAF) <0.01 were excluded. Healthy controls were genotyped using the Illumina Infinium CoreExome genotyping array. QC was consistent with that described above for JIA samples. Suggestive signals were followed-up for meta-analysis with a previous GWAS (2751 cases/15 886 controls). We tested for enrichment of association signals in a broad range of functional annotations, and integrated statistical fine-mapping and experimental data to identify target genes.  GWAS SUMMARY DATA FOR JIAU  The author performed data collection, quality control, and statistical analyses in 2 phases, and then jointly analyzed the data from both phases to improve the power of locus discovery. Samples for genotyping were collected from 2 cohorts: 1) a Dutch cohort, comprising 137 patients with JIA-associated uveitis and 247 non-uveitis JIA control patients (phase 1), and 2) a cohort of samples collected in Germany, Belgium, and Switzerland, comprising 77 patients with JIA-associated uveitis and 115 non-uveitis JIA controls (phase 2).  GWAS SUMMARY DATA FOR GUT MICROBIOTA  The participants' 16S ribosomal RNA gene sequences and genotyped data were analyzed to investigate the relationship between human autosomal genetic variation and gut microbial communities. |
|  | c) | Describe measurement, quality control and selection of genetic variants | 4,5 | GWAS SUMMARY DATA FOR JIA  Identity-by-descent was used to identify related individuals across all study samples. For each related pair, the sample with the highest call rate was retained. Outliers were identified and excluded based on ancestry using principal component (PC) analysis performed with the flashpca software package (V.2.0) where outliers were identified using aberrant R library (V.1.0). The total number of individuals that remained in the final QC-filtered data set was 12 501 (3305 cases and 9196 healthy controls). The QC-filtered GWAS data set was subjected to whole-genome genotype imputation. Haplotype phasing and imputation were performed in the Michigan Imputation server using SHAPEIT214 and Minimac respectively, and the Haplotype Reference Consortium reference panel. Following imputation, SNPs were excluded based on MAF <0.01 and imputation quality (r^2^) <0.4. Case-control association testing was performed by SNPTEST software package (V.2.5.2). Three PCs were included as covariates to account for any residual population substructure. Any SNP with a p value <5 x 10^-6^ was selected for validation in GWAS summary statistics from an independent data set of 2751 JIA cases (oligoJIA and RF–polyJIA) and 15 886 controls of European ancestry. An inverse variance weighted fixed effects meta-analysis was performed using the software package GWAMA (V.2.2.2). The presence of heterogeneity of ORs across data sets was evaluated with the test statistics I^2^ and Q.  GWAS SUMMARY DATA FOR JIAU  The author genotyped all samples on the Infinium HumanOmniExpress-24 array (version 1.1). In each phase, they performed sample- and variant-level quality control, prephasing, and imputation. We prephased the phase 1 and phase 2 data separately using the ShapeIt2 program. As the sample size was >100 samples, they ran prephasing without a reference panel, in accordance with the ShapeIt2 recommendations. Following prephasing, the author imputed the prephased samples using Impute2 software, and an imputation reference panel was constructed using the 2,504 samples that were whole-genome sequenced in the 1000 Genomes Project (phase 3) . Within each study phase, they performed a GWAS, using Plink software version 1.9 24, in an additive logistic regression model, with corrections for sex and the first 2 principal components (PCs; to account for sample ancestry). The author meta-analyzed the results using the METAL program 25 for a combined analysis of 192 patients with JIA-associated uveitis and 330 non-uveitis JIA controls (Table 1). To ensure that we were analyzing SNPs with high-quality imputation, the author only analyzed common SNPs (i.e., those with a minor allele frequency of >1%) with an imputation quality (info) score of >0.7. The single signal identified as achieving genome-wide significance (P < 2 × 10^−8^, accounting for 3 phenotype comparisons) resided in the MHC.  GWAS SUMMARY DATA FOR GUT MICROBIOTA  The participants' 16S ribosomal RNA gene sequences and genotyped data were analyzed to investigate the relationship between human autosomal genetic variation and gut microbial communities. The study included 211 taxa comprising 35 families, 20 orders, 16 phyla, 9 orders, and 131 genera. |
|  | d) | For each exposure, outcome, and other relevant variables, describe methods of assessment and diagnostic criteria for diseases | 4 | The GWAS data for JIA is derived from a meta-analysis of 6,056 JIA patients and 25,086 European ancestry controls recently updated. The diagnosis of JIA patients follows the international standards published by the International League of Associations for Rheumatology (ILAR). The JIA GWAS data systematically analyzed the specificity and shared susceptibility loci for different ILAR subtypes of JIA. The GWAS data for JIAU is sourced from the study by Haasnoot et al., which included 192 JIAU patients of European ancestry and 330 JIA control patients without uveitis. |
|  | e) | Provide details of ethics committee approval and participant informed consent, if relevant | 7 | Standard Protocol Approval, Registration and Patient Consent  The GWAS data used in this study were all from publicly available databases. The summary statistics of JIA, JIAU and gut microbiota abundance do not contain any personal information, and each GWAS has received ethical approval from the relevant ethical review board. |
| 5 | **Assumptions** | Explicitly state the three core IV assumptions for the main analysis (relevance, independence and exclusion restriction) as well assumptions for any additional or sensitivity analysis | 4 | STUDY DESIGN  The aim of this study was to evaluate the causal relationship between genetically predicted JIA/JIAU risk and genetically predicted abundance of gut microbiota using a Mendelian randomization method. The Mendelian randomization design consisted of three components. Firstly, the selection of genetic variants as instrumental variables for JIA/JIAU. Secondly, the acquisition of a summary dataset for genetic instruments derived from a genome-wide association study of JIA/JIAU, and finally, obtaining a summary dataset for single nucleotide polymorphism results. These results were used to investigate the impact of GWAS genetic instruments on gut microbiota. Fig. 1 outlines the design of the Mendelian randomization study, while Fig. 2 presents an overview of the investigation along with a flow chart. |
| 6 | **Statistical methods: main analysis** | Describe statistical methods and statistics used |  |  |
|  | a) | Describe how quantitative variables were handled in the analyses (i.e., scale, units, model) | 6 | The initial analysis utilized inverse variance weighting (IVW) to explore the possible causal relationship between gut microbiota abundance and JIA/JIAU. |
|  | b) | Describe how genetic variants were handled in the analyses and, if applicable, how their weights were selected | 5,6 | SELECTION OF INSTRUMENTAL VARIABLES  This study aimed to explore the causal relationship between gut microbiota and JIA/JIAU through the Mendelian Randomization analysis of instrumental variables. Given the limited number of genetic variants associated with gut microbiota, to ensure an adequate number of candidate instruments for the forward MR analysis, we set the significance threshold at *p* < 1.0×10^-5^ based on reference to the majority of MR studies on gut microbiota. In addition, to ensure the independence between iIVs and avoid bias caused by linkage disequilibrium (LD) between SNPs, we conducted clustering with a threshold of r^2^ < 0.01 and a clustering distance of 500 kb to evaluate LD. Finally, we removed IVs with an F statistic below 10 to minimize potential weak instrument bias. The F statistic is calculated as (where N represents the sample size and R2 refers to the squared correlation coefficient).  For reverse MR, the significance threshold was chosen at p < 5.0×10^-6^ to screen SNPs as IVs for JIA and p < 1.0×10^-5^ for JIAU (LD: r2 < 0.001, and clumping distance = 10,000 kb). |
|  | c) | Describe the MR estimator (e.g. two-stage least squares, Wald ratio) and related statistics. Detail the included covariates and, in case of two-sample MR, whether the same covariate set was used for adjustment in the two samples | 6 | Two-Sample Mendelian randomization  The initial analysis utilized inverse variance weighting (IVW) to explore the possible causal relationship between gut microbiota abundance andJIA/JIAU.  Additionally, for mendelian randomization analysis, the weighted median, MR-Egger, simple mode, and weighted mode methods were used. |
|  | d) | Explain how missing data were addressed | 4,5 | Identity-by-descent was used to identify related individuals across all study samples. For each related pair, the sample with the highest call rate was retained. Outliers were identified and excluded based on ancestry using principal component (PC) analysis performed with the flashpca software package (V.2.0) where outliers were identified using aberrant R library (V.1.0). The total number of individuals that remained in the final QC-filtered data set was 12 501 (3305 cases and 9196 healthy controls). The QC-filtered GWAS data set was subjected to whole-genome genotype imputation. Haplotype phasing and imputation were performed in the Michigan Imputation server using SHAPEIT214 and Minimac respectively, and the Haplotype Reference Consortium reference panel. Following imputation, SNPs were excluded based on MAF <0.01 and imputation quality (r^2^) <0.4. Case-control association testing was performed by SNPTEST software package (V.2.5.2). Three PCs were included as covariates to account for any residual population substructure. Any SNP with a p value <5 x 10^-6^ was selected for validation in GWAS summary statistics from an independent data set of 2751 JIA cases (oligoJIA and RF–polyJIA) and 15 886 controls of European ancestry. An inverse variance weighted fixed effects meta-analysis was performed using the software package GWAMA (V.2.2.2). The presence of heterogeneity of ORs across data sets was evaluated with the test statistics I^2^ and Q. |
|  | e) | If applicable, indicate how multiple testing was addressed | 6 | A multiple test significance threshold was set at 0.05/n (where n represents the number of independent bacterial taxa at the corresponding taxonomic level) due to the numerous comparisons that took place at each character level, such as phylum, class, order, family, and genus. Significance values that fell between the multiple test significance threshold and 0.05 were considered potentially significant. |
| 7 | **Assessment of assumptions** | Describe any methods or prior knowledge used to assess the assumptions or justify their validity | 6 | Given the limited number of genetic variants associated with gut microbiota, to ensure an adequate number of candidate instruments for the forward MR analysis, we set the significance threshold at p < 1.0×10^-5^ based on reference to the majority of MR studies on gut microbiota. In addition, to ensure the independence between iIVs and avoid bias caused by linkage disequilibrium (LD) between SNPs, we conducted clustering with a threshold of r^2^ < 0.01 and a clustering distance of 500 kb to evaluate LD. For reverse MR, we set the significance level to *P*<5.0×10-6, LD r^2^<0.001, and clumping distance=10,000 kb to screen SNPs as IVs for JIA and JIAU. Finally, we removed IVs with an F statistic below 10 to minimize potential weak instrument bias. The F statistic is calculated as (where N represents the sample size and R^2^ refers to the squared correlation coefficient). Meanwhile, those having a F-statistic >10 are included for further analysis. |
| 8 | **Sensitivity analyses and additional analyses** | Describe any sensitivity analyses or additional analyses performed (e.g. comparison of effect estimates from different approaches, independent replication, bias analytic techniques, validation of instruments, simulations) | 7 | We conducted several sensitivity analyses consisting of tests such as Cochran's Q statistic, funnel plots, leave-one-out analysis, and the MR-Egger intercept test. Cochran's Q test revealed heterogeneity in the instrumental variables in case the p-value was lower than 0.05. The "leave-one-out" method was applied to validate the causal relationship between JIA/JIAU and gut microbiota abundance.  For detecting and correcting pleiotropic outliers, we employed the mendelian randomized pleiotropic residuals and outliers (MR-PRESSO) method. |
| 9 | **Software and pre-registration** |  |  |  |
|  | a) | Name statistical software and package(s), including version and settings used | 6 | Statistical analyses were performed using R software version 4.2.2, utilizing the R packages "TwoSampleMR" (v.0.5.6), "MRPRESSO" (v.1.0), and "MendelianRandomization"(OYJS, 2023) (v.0.7.0) in order to carry out a Mendelian randomization (MR) analysis on the causal relationship between JIA/JIAU and gut microbiota. |
|  | b) | State whether the study protocol and details were pre-registered (as well as when and where) |  | This study protocol and details were not pre-registered. |
|  | **RESULTS** |  |  |  |
| 10 | **Descriptive data** |  |  |  |
|  | a) | Report the numbers of individuals at each stage of included studies and reasons for exclusion. Consider use of a flow diagram |  | The GWAS data used in this study is derived from the GWAS data of existing studies, so it is not reported. |
|  | b) | Report summary statistics for phenotypic exposure(s), outcome(s), and other relevant variables (e.g. means, SDs, proportions) |  | We report summary statistics for JIA, JIAU, and gut microbiota data in Supplementary Table S1. |
|  | c) | If the data sources include meta-analyses of previous studies, provide the assessments of heterogeneity across these studies |  | Not applicable. |
|  | d) | For two-sample MR:  i.  Provide justification of the similarity of the genetic variant-exposure associations between the exposure and outcome samples  ii.  Provide information on the number of individuals who overlap between the exposure and outcome studies | 7,8 | **Forward MR**  A total of 3036 SNPs relevant to gut microbiota at the phylum, class, order, family, and genus levels were identified in the first step. Each SNP had an F statistic ＞ 10, suggesting the absence of weak instrumental bias (Table S2).  Additionally, the steiger filtering analysis results of IVs for gut microbiota on JIA found no SNPs with a reverse causal direction. (Table S3). However, in the case of JIAU, the steiger filtering analysis helped eliminate 849 SNPs that exhibited reverse causal directions (Table S5). For our analysis, we identified 131 genera, 35 families, 16 classes, 20 orders, and 9 phyla as IVs. There were 5 to 26 IVs obtained from each classification. **Reverse MR**  For JIA, 13 SNPs fulfilled the IVs screening requirements, while for JIAU, 6 SNPs met the criteria. All of these SNPs had an F-statistics ＞ 10, representing the lack of weak instrumental bias (see Table S13 and Table S19). Additionally, There were no SNPs found using the Steiger filtering technique that had opposing causal orientations (Table S14 and Table S20). |
| 11 | **Main results** |  |  |  |
|  | a) | Report the associations between genetic variant and exposure, and between genetic variant and outcome, preferably on an interpretable scale |  | Table S2, Table S13, Table S19, |
|  | b) | Report MR estimates of the relationship between exposure and outcome, and the measures of uncertainty from the MR analysis, on an interpretable scale, such as odds ratio or relative risk per SD difference |  | Table S4, Table S6, Table S15, Table S21 |
|  | c) | If relevant, consider translating estimates of relative risk into absolute risk for a meaningful time period |  | Not applicable. |
|  | d) | Consider plots to visualize results (e.g. forest plot, scatterplot of associations between genetic variants and outcome versus between genetic variants and exposure) |  | Figure S1, S5, S9 and S13 are scatter plot of associations between genetic variants and outcome versus between genetic variants and exposure;  Figure S2, S6 ,S10 and S14 are forest plot of associations between genetic variants and outcome versus between genetic variants and exposure. |
| 12 | **Assessment of assumptions** |  |  |  |
|  | a) | Report the assessment of the validity of the assumptions | 7 | 17 SNPs met the instrumental variable screening criteria for nicotine dependence, and all had an F-statistic >10 indicating no weak instrumental bias (Table S2). |
|  | b) | Report any additional statistics (e.g., assessments of heterogeneity across genetic variants, such as *I^2^*, Q statistic or E-value) | 9 | No evidence was found for horizontal pleiotropy when using the MR-Egger regression intercept method on the gut microbiota and nicotine-dependent instrumental variables (Table S12, Table S13 and Table S14). We screened and removed any outliers in the MR-PRESSO analysis and found no horizontal pleiotropy for the gut microbiota or nicotine-dependent instrumental variables (Table S15 and Table S16). Furthermore, the majority of Cochrane Q statistics did not show significant heterogeneity (*p* > 0.05) as shown in the Supplementary Material (Table S9, Table S10 and Table S11). In cases where heterogeneity was found to be significant, we used a random-effects model with an IVW model. |
| 13 | **Sensitivity analyses and additional analyses** |  |  |  |
|  | a) | Report any sensitivity analyses to assess the robustness of the main results to violations of the assumptions |  | In order to enhance the robustness of the analysis, we have supplemented Mendelian randomization (MR) results using four methods: MR Egger, Simple mode, Weighted median, Weighted mode, as shown in Supplementary Tables S4, S6, S15 and S21. |
|  | b) | Report results from other sensitivity analyses or additional analyses |  | We have provided the results of Mendelian randomization using the MR-PRESSO method in Supplementary Tables S9 S12 and S18. |
|  | c) | Report any assessment of direction of causal relationship (e.g., bidirectional MR) | 7 | Reverse Mendelian randomization analysis  To investigate whether JIA/JIAU was associated with altered gut flora abundance, we performed reverse Mendelian randomization (MR) analyses using SNPs associated with JIA/JIAU as instrumental variables (JIA/JIAU was the exposure and gut flora was the outcome). |
|  | d) | When relevant, report and compare with estimates from non-MR analyses | 10 | Previous research has confirmed that the microbial composition in JIA patients is different from that of healthy children, such as an increase in phyla Bacteroidetes and a notable reduction in Firmicutes in pediatric patients diagnosed with JIA. This study suggests an increase in the content of class betaproteobacteria in JIA, which is consistent with previous research findings. The results of the MR study suggest an increase in the abundance of genus Phascolarctobacterium in JIAU, contrary to the previous study results. On one hand, this might be due to JIAU as a complication of JIA, with a different impact on the gut microbiota, and on the other hand, it further supports the notion that eenvironmental stimuli exert a stronger influence. This study found a rise in the level of genus Roseburia in JIA, which contradicts previous research results. |
|  | e) | Consider additional plots to visualize results (e.g., leave-one-out analyses) |  | We have provided the results of the Leave-one-out analysis in Supplementary Figures S3, S7 S11 and S15. Additionally, the analysis results of funnel plots are presented in Supplementary Figures S4, S8, S12 and S16. |
|  | **DISCUSSION** |  |  |  |
| 14 | **Key results** | Summarize key results with reference to study objectives | 9 | Our study employed a bidirectional Mendelian randomization approach to assess the causality between JIA/JIAU and gut microbial abundance. To our knowledge, this is the first mendelian randomization study to examine the causal relationship between JIA/JIAU and gut microbial abundance. |
| 15 | **Limitations** | Discuss limitations of the study, taking into account the validity of the IV assumptions, other sources of potential bias, and imprecision. Discuss both direction and magnitude of any potential bias and any efforts to address them | 11,12 | While our study has several advantages, we also recognize certain drawbacks. Firstly, when compared to the Bonferroni technique corrected for importance, the p-values in our analysis are not as strong. Nevertheless, that our research is driven by hypotheses and supported by substantial biological data and past studies support the epidemiological link between gut microbiota and JIA or JIAU, future research may need to include samples from a larger population of JIA and JIAU patients to further strengthen this result. Secondly, adjusting p-values through numerous comparisons may raise the possibility of unfavorable outcomes and may weaken the number and multi-level structure of microbial communities (abundance and correlation between microbial strains) as well as the correlation between JIA or JIAU. As a result, caution needs to be used when interpreting unfavorable outcomes or potentially significant p-values. Furthermore, the two-sample MR method is a theoretical causal analysis method, and it can be seen that the conclusions of our study have many contradictions with epidemiological studies, which also suggests the impact of environmental risk factors on JIA or JIAU. In addition, since the specific mechanisms of the gut microbiome in the onset of JIA or JIAU are still unclear, further research, including animal experiments, is needed to verify the results of the MR studies and causality. Finally, since the majority of JIA group participants are European in origin, the results we obtained may not be as applicable to other ethnic communities as we hope. |
| 16 | **Interpretation** |  |  |  |
|  | a) | Meaning: Give a cautious overall interpretation of results in the context of their limitations and in comparison with other studies | 11 | Specifically, we employed a biodirectional mendelian randomization analysis to establish the causal association between gut microbiota and JIA/JIAU. This approach allowed us to control for confounding factors and minimize the risk of reverse causation. The gut microbiota and JIA/JIAU genome-wide association data were retrieved from the largest available GWAS meta-analysis to ensure the statistical robustness of the instrumental variables used in the Mendelian randomization analysis. To minimize the potential impact of weak IV bias, we employed a suitable threshold for the genomic correlation of instrumental variables (*p* = 1×10^-5^). This threshold was chosen based on the availability of a sufficient number of SNPs with adequate statistical power for most gut flora, effectively avoiding confounding.However, there was no way to compare our study with previous studies because there were no previous MR analyses on gut flora and JIA/JIAU. |
|  | b) | Mechanism: Discuss underlying biological mechanisms that could drive a potential causal relationship between the investigated exposure and the outcome, and whether the gene-environment equivalence assumption is reasonable. Use causal language carefully, clarifying that IV estimates may provide causal effects only under certain assumptions | 9,10 | The intestinal microbiota mainly contributes to the pathogenesis of JIA by altering the intestinal mucosal permeability and regulating the host immune system . Previous research has confirmed that the microbial composition in JIA patients is different from that of healthy children, such as an increase in phyla Bacteroidetes and a notable reduction in Firmicutes in pediatric patients diagnosed with JIA. This study suggests an increase in the content of class betaproteobacteria in JIA, which is consistent with previous research findings. The results of the MR study suggest an increase in the abundance of genus Phascolarctobacterium in JIAU, contrary to the previous study results. On one hand, this might be due to JIAU as a complication of JIA, with a different impact on the gut microbiota, and on the other hand, it further supports the notion that eenvironmental stimuli exert a stronger influence. This study found a rise in the level of genus Roseburia in JIA, which contradicts previous research results. |
|  | c) | Clinical relevance: Discuss whether the results have clinical or public policy relevance, and to what extent they inform effect sizes of possible interventions | 10 | Based on the important role of gut microbiota in arthritis, targeted probiotics are gradually becoming a new treatment option in the context of rheumatic diseases. Since the mature and stable state of the microbiota once formed is difficult to change, and childhood is a critical period to acquire basic functions (such as immune tolerance to commensal microbiota), these findings present a unique opportunity to intervene and potentially modify the disease progression by targeting the microbiota, thereby offering a valuable time window for effective interventions. The use of prebiotics and probiotics in clinical trials is on the rise, with an expanding body of evidence showing favorable tolerance and possible advantages that assist the microbiota of infants return to health. Nevertheless, the randomized controlled trial of probiotics by Anuj Shukl et al. showed good tolerance in patients with Enthesitis-related arthritis (ERA), but did not demonstrate any appreciable clinical or immunological effects as compared to to non-steroidal anti-inflammatory medication therapy. Therefore, in order to assess efficacy and safety of probiotics for JIA, further clinical data is required, providing better recommendations for clinical practice. |
| 17 | **Generalizability** | Discuss the generalizability of the study results (a) to other populations, (b) across other exposure periods/timings, and (c) across other levels of exposure | 12 | Since the majority of JIA group participants are European in origin, the results we obtained may not be as applicable to other ethnic communities as we hope. |
|  | **OTHER INFORMATION** |  |  |  |
| 18 | **Funding** | Describe sources of funding and the role of funders in the present study and, if applicable, sources of funding for the databases and original study or studies on which the present study is based |  | Funding information  This research was funded by Department of Science and Technology of Guangdong Province, Efficacy and safety of the Jianer Jiedu Formula for the treatment of novel coronavirus infections in children--a real-world and randomized controlled study (No. 2023B1111020004), and State Administration of Traditional Chinese Medicine, a project for Chinese Medicine on Ying Lv's Renowned Expert Inheritance Studio (No. E43729). These funding organizations did not participate in the study design, data collection, analysis, interpretation or writing of the manuscript or in the decision to submit the manuscript for publication. |
| 19 | **Data and data sharing** | Provide the data used to perform all analyses or report where and how the data can be accessed, and reference these sources in the article. Provide the statistical code needed to reproduce the results in the article, or report whether the code is publicly accessible and if so, where |  | Generated Statement: Publicly available datasets were analyzed in this study. This data can be found here: The GWAS meta-analysis results on JIA can be downloaded from <https://www.ncbi.nlm.nih.gov/pmc/articles/PMC7892389/.>The summary data on JIAU can be downloaded from <https://github.com/saralpulit/UveitisJIA_MHC-fineMapping.> The summary data on gut microbiota is from MiBioGen consortium, which can be obtained from the IEU GWAS database (https://gwas.mrcieu.ac.uk/) (GWAS ID: ebi-a-GCST90016908-- ebi-a-GCST90017118). |
| 20 | **Conflicts of Interest** | All authors should declare all potential conflicts of interest |  | Conflict of interest statement  The researchers affirm that there were no commercial or financial affiliations that could be considered a potential conflict of interest during the conduct of the study. |

This checklist is copyrighted by the Equator Network under the Creative Commons Attribution 3.0 Unported (CC BY 3.0) license.

1. Skrivankova VW, Richmond RC, Woolf BAR, Yarmolinsky J, Davies NM, Swanson SA, et al. Strengthening the Reporting of Observational Studies in Epidemiology using Mendelian Randomization (STROBE-MR) Statement. JAMA. 2021;under review.

2. Skrivankova VW, Richmond RC, Woolf BAR, Davies NM, Swanson SA, VanderWeele TJ, et al. Strengthening the Reporting of Observational Studies in Epidemiology using Mendelian Randomisation (STROBE-MR): Explanation and Elaboration. BMJ. 2021;375:n2233.
